# Modeling the assembly order of multimeric heteroprotein complexes

**DOI:** 10.1371/journal.pcbi.1005937

**Published:** 2018-01-12

**Authors:** Lenna X. Peterson, Yoichiro Togawa, Juan Esquivel-Rodriguez, Genki Terashi, Charles Christoffer, Amitava Roy, Woong-Hee Shin, Daisuke Kihara

**Affiliations:** 1 Department of Biological Sciences, Purdue University, West Lafayette, Indiana, United States of America; 2 Department of Computer Science, Purdue University, West Lafayette, Indiana, United States of America; 3 Department of Medicinal Chemistry and Molecular Pharmacology, Purdue University, West Lafayette, Indiana, United States of America; 4 Bioinformatics and Computational Biosciences Branch, Rocky Mountain Laboratories, NIAID, National Institutes of Health, Hamilton, Montana, United States of America; Institute for Research in Biomedicine, SPAIN

## Abstract

Protein-protein interactions are the cornerstone of numerous biological processes. Although an increasing number of protein complex structures have been determined using experimental methods, relatively fewer studies have been performed to determine the assembly order of complexes. In addition to the insights into the molecular mechanisms of biological function provided by the structure of a complex, knowing the assembly order is important for understanding the process of complex formation. Assembly order is also practically useful for constructing subcomplexes as a step toward solving the entire complex experimentally, designing artificial protein complexes, and developing drugs that interrupt a critical step in the complex assembly. There are several experimental methods for determining the assembly order of complexes; however, these techniques are resource-intensive. Here, we present a computational method that predicts the assembly order of protein complexes by building the complex structure. The method, named Path-LzerD, uses a multimeric protein docking algorithm that assembles a protein complex structure from individual subunit structures and predicts assembly order by observing the simulated assembly process of the complex. Benchmarked on a dataset of complexes with experimental evidence of assembly order, Path-LZerD was successful in predicting the assembly pathway for the majority of the cases. Moreover, when compared with a simple approach that infers the assembly path from the buried surface area of subunits in the native complex, Path-LZerD has the strong advantage that it can be used for cases where the complex structure is not known. The path prediction accuracy decreased when starting from unbound monomers, particularly for larger complexes of five or more subunits, for which only a part of the assembly path was correctly identified. As the first method of its kind, Path-LZerD opens a new area of computational protein structure modeling and will be an indispensable approach for studying protein complexes.

## Author summary

Protein-protein interactions, particularly those involving multiple proteins, are the cornerstone of numerous biological processes. Although an increasing number of multi-chain protein complex structures have been determined, fewer studies have been performed to determine the assembly order of complexes. Knowing the assembly order of a complex provides insights into the process of complex formation. Assembly order is also practically useful for reconstructing and determining the structure of a subcomplex of a large protein complex. It also has important applications including designing artificial protein complexes and drugs that prevent the assembly of protein complexes. We present a computational method, Path-LZerD, which predicts the assembly order of a protein complex by simulating its assembly process. This is the first method of this kind. A strong advantage of Path-LZerD is that the assembly order can be predicted even when the overall complex structure is not known. Path-LZerD opens a new area of computational protein structure modeling and will be an indispensable approach for studying protein complexes.

## Introduction

Many biological processes involve protein complexes with multiple subunits. Insights into the molecular mechanisms of the functions of these multimeric complexes can be gleaned from their quaternary structures, which are determined by experimental methods including X-ray crystallography [1], nuclear magnetic resonance (NMR) [2, 3], small-angle X-ray scattering (SAXS) [4], and electron microscopy [5]. Computational methods have been also used for modeling protein complexes [6–10].

Although an increasing number of protein complex structures have been revealed, there has been relatively less work conducted to elucidate the mechanisms of protein complex assembly: in particular, the assembly order of protein complexes. Tompa and Rose [11] discussed in the context of interactions of the whole proteome that the assembly of the interactome has an enormous number of combinations, which makes random exploration unrealistic, analogous to the Levinthal paradox [12]. They concluded that there must be hierarchical assembly pathways that make the correct formation of individual complexes possible. Ordered pathways allow efficient assembly and may reduce the possibility of forming incorrect topology. Many protein complexes have evolved to assemble in a defined order as shown in gene fusion events [13, 14] or conserved gene orders [15]. Some complexes are required to follow an ordered assembly pathway for realizing their biological functions. For example, in ATP synthase, the proton channel forms as the last step which avoids the negative consequences of futile proton transport [16]. Thus, the order of subunit assembly can offer critical clues to the function and evolution of a multimeric complex.

From a practical standpoint, knowledge of assembly order is helpful for in vitro reconstitution of multimeric protein complexes. When solving the entire complex is difficult, knowledge of the assembly order can allow reconstruction of a subcomplex which may be easier to solve. Assembly order needs to be taken into account when designing artificial protein complexes [17]. In addition, designing drugs that target protein-protein interactions is of increasing interest [18], and knowledge of the assembly order is indispensable for creating and evaluating drugs that prevent a critical step in a protein complex assembly pathway [19].

The assembly order of a multimeric complex can be experimentally determined by reconstructing stable intermediates of two or more subunits [20], which are detected, for example, by gel electrophoresis [21, 22] or co-immunoprecipitation [23]. Real-time mass spectrometry can identify stable subcomplexes that appear in the assembly time course [24, 25]. Deletion mutants were constructed to examine if deletions affect complex assembly [26]. Pulse-chase monitored by quantitative mass spectrometry (PC/QMS) was applied to investigate assembly pathways for the 30s ribosomal subunit, which detects an assembly order by measuring the ratio of labeled and unlabeled proteins that are added later in the time course [27]. Recently, single-particle electron microscopy was used to determine subcomplex structures stained at different time points of assembly in combination with mass spectrometry [28, 29]. Alternatively, assuming that assembly and disassembly proceed via the same pathway in opposite directions, electrospray ionization mass spectrometry (ESI-MS) can be used to determine disassembly pathways [30].

Previous works by Teichmann and her colleagues used the buried surface area (BSA) of each subunit to predict the complex assembly order [13, 14]. BSA is the difference between the solvent-accessible surface area (SASA) of a subunit in the complex and in the isolated state. From thermodynamics principles, the assembly order of a multimeric protein complex is determined probabilistically by the population sizes of various subcomplexes that appear during the assembly process, where the population size of each subcomplex is determined by its binding free energy. BSA was used in their works as a rough approximation of binding free energy, where large buried surface area corresponds to lower (more favorable) binding free energy and thus earlier assembly. The BSA method agreed with experimentally determined assembly order in thirteen out of sixteen (81.3%) homomeric protein complexes [13] and seven out of nine (77.8%) heteromeric cases [14]. However, the BSA method has two primary limitations. BSA has been found to have only moderate correlation to binding free energy [31, 32]. More fundamentally, the BSA method requires a complete protein complex structure; thus, it cannot be applied to cases where all subunits of the complex have been solved separately but a complete structure is not available.

In this work, we used a multiple protein docking method, Multi-LZerD [33], developed in our group, which can simulate the assembly process of protein complexes to predict the docking order of protein complexes [34]. Multi-LZerD builds structure models of a multimeric protein complex from the structures of its individual subunits. The complex structure model is assembled by combining pairwise docking models of subunits, which are predicted by a pairwise protein docking program, LZerD [35, 36]. Complex models are refined in many generations of a genetic algorithm, which finally produces about 200 models. The entire Multi-LZerD algorithm of producing multimeric complex structure models somewhat mimics the actual complex assembly procedure; in fact, it was found that the assembly order of complexes can be well predicted by analyzing the assembly pathways of models produced and refined in the Multi-LZerD model building process. The key observation that led to the path prediction is that the binding energy of pairwise docking models assembled to construct a complex indicates the docking order. The method to predict complex assembly order with Multi-LZerD is called Path-LZerD.

A strong advantage of Path-LZerD is that, unlike the BSA method, the assembly order can be predicted even when the complex structure is not determined yet, because with Path-LZerD the assembly order of a multimeric complex is predicted by simulating the assembly process of the complex. The binding free energy of subunits was estimated using knowledge-based statistical contact potentials, which can evaluate the energy more accurately than simply considering BSA and are successful in protein-protein docking [37]. Interestingly, in many cases the assembly order was correctly predicted even when the predicted structure models from Multi-LZerD were not entirely correct. Using 21 protein complexes with between three and seven subunits, the complex structure and/or topology was well predicted in nine cases and the assembly order was correctly predicted for ten cases. When homology models or unbound structures were used for building complex structures, the assembly order was similarly well predicted for small complexes of 3 and 4 subunits; however, the predictions deteriorated for larger complexes. The ability to predict assembly order from the structures of the subunits can offer additional insights into the biological function of multimeric protein complexes.

## Materials and methods

We will first describe the dataset of multimeric protein complexes used to evaluate Path-LZerD. Then, we will briefly introduce the Multi-LZerD multiple protein docking program, which is the core of the assembly order prediction. The scoring functions used are also explained. Then, we explain how assembly orders were predicted using Path-LZerD and the rationale behind the strategy.

### Dataset

The dataset of multimeric protein complexes includes 21 complexes of 3-7 chains, which have evidence of their complex assembly order (Table 1). The set was manually collected from literature and from the Protein Data Bank (PDB) [38]. The assembly pathway of each protein complex is listed in Table 1. For example, the assembly order of BG> BGP for 1a0r indicates that chain B and G form a subcomplex first, to which chain P docks to construct the complex. A chain ID with superscript prime (′) or a number, for example, B′ or B^4^, indicates that it has the same sequence as chain B. The evidence for assembly pathways is classified into four categories shown in the last column of the table: experimental evidence (E), biological inference (B), structural inference (S), and model of assembly (M). “Experimental evidence” includes co-immunoprecipitation of subcomplexes and ESI-MS. “Biological inference” indicates that the order of the assembly can be reasonably inferred from the function of each subunit. For example, if the complex is between a protein dimer and its inhibitor, the dimer is expected to form first and the inhibitor to bind later. Another type of evidence, “structural inference,” is from structural information of a complex, which indicates that features of the structure, such as which chains are in contact, restrict the possible assembly orders. “Model of assembly” indicates that the assembly pathway has been proposed in a publication. A detailed explanation of the evidence for the assembly order of each complex is provided in S1 Appendix. The assembly order prediction was performed for bound and unbound/computationally modeled cases of this set of proteins.

**Table 1:** List of multimeric complexes used for prediction.

N	PDB	Name	Pathway(s)	Evidence
3	1a0r	Transducin *βγ* dimer bound to phosducin	BG> BGP	E,B
	1ikn	I-*κ*-B *α*/NF-*κ*-B complex	AC> ACD	E,B,M
	1vcb	ElonginBC bound to VHL	AB> ABC	E,B,M
	2aze	Rb C-terminal bound to E2F1-DP1	AB> ABC	E,B
4	1es7	Complex between BMP-2 and 2 BMP receptors	AA′> AA′B> AA′BB′	S
	1gpq	IVY complex with its target HEWL	AA′> AA′C> AA′CC′	B,S
	2e9x	Human GINS core complex	BD> ABD> ABCD	E
	1kf6	Fumarate reductase	CD> BCD> ABCD	E,M
	2bq1	Ribonucleotide reductase	EE′+II′> EE′II′	E,B,S
	2qsp	Bovine hemoglobin at pH 5.7	AB> AB+A′B′> AA′BB′	E,M
	3fh6	Maltose transporter	AA′> AA′F *or* AA′> AA′G *or* AA′+FG> AA′FG	E,M
5	1hez	Antibody-antigen complex	AB> AB+A′B′> AB+A′B′E> AA′BB′E	B,S
	1w88	Pyruvate dehydrogenase E1 bound to a subunit of E2	AA′BB′> AA′BB′I	B
6	1du3	TRAIL-SDR5	DD′> DD′D″> ADD′D″> AA′DD′D″> AA′A″DD′D″	B,S
	1rlb	Retinol binding protein bound to transthyretin	AA′> AA′+A″A‴> AA′A″A‴> AA′A″A‴E> AA′A″A‴EE′	B,S
	1s5b	Cholera holotoxin with an A-subunit	B^1^B^2^> B^1^B^2^B^3^> AB^1^B^2^B^3^> AB^1^B^2^B^3^B^4^> AB^1^B^2^B^3^B^4^B^5^^†^	E,M
	3vyt	HypCDE complex	CD+C′D′+EE′> CD+C′D′EE′> CC′DD′EE′^†^	E,M
	4hi0	UreF/UreH/UreG complex	FH+F′H′+GG′> FF′HH′+GG′> FF′GG′HH′^†^	E,M
	4igc	Bacterial RNA polymerase	AA′> AA′C> AA′C+DE> AA′CDE> AA′CDEX	E,M
7	3uku	Arp2/3	CG+DF> ACDFG> ABCDEFG	E,M
	4gwp	Mediator head module	ABD> ABCDG+EF> ABCDEFG	E,M

N: Number of chains. PDB: PDB ID of bound complex structure. Pathways marked with dagger (†) use letters corresponding to protein names; others use PDB chain IDs. Evidence types: E: experimental evidence; B: biological inference; S: structural inference; M: model of assembly.

### Multi-LZerD

**Fig 1:**
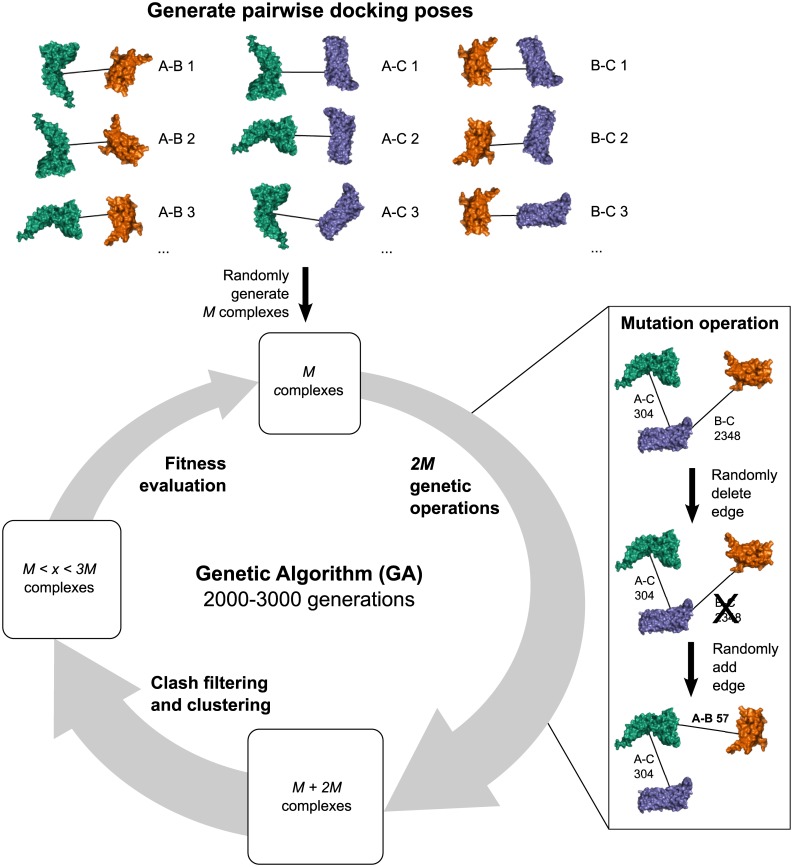
Overview of the Multi-LZerD algorithm. Here, an example of a 3-chain complex is shown. The first step is to generate pairwise docking poses (decoys) with LZerD for each pair, A-B, A-C, and B-C, which are ranked by a scoring function. Usually about a few thousand poses are kept for each pair (top panel). Then, the Multi-LZerD population is initialized by generating *M* random complexes. A complex is represented as a spanning tree, where each node is a protein chain and each edge is a pairwise decoy. The first complex in the right panel is composed of 304th decoy ranked by the score between A and C and 2348th decoy between B and C. 2*M* mutation operations are performed to increase the population size and variation (right panel). A mutation involves deleting a random edge and adding a random edge. Next, the population is filtered for clashes and clustered. Finally, the top *M* complexes by the molecular mechanics score are kept, concluding one generation. This process is repeated for 2000 generations. If the population has not converged, another 1000 generations are run.

The assembly pathway prediction by Path-LZerD uses the Multi-LZerD [33] algorithm at the core of its protocol. Multi-LZerD predicts the structure of a multimeric protein complex from the structures of the subunits of the complex (Fig 1). In the first step, all pairwise combinations of subunits are docked using a pairwise protein docking method, LZerD [6, 35, 39, 40]. LZerD represents protein surface shape using 3D Zernike descriptors (3DZD) [39, 41, 42], which are based on a mathematical series expansion of a 3D function (in this case, protein surface shape). The 3DZD are a soft representation of the surface shape, conferring tolerance to the conformational changes associated with binding. Typically, over 100,000 docking models (decoys) are generated for a pair of protein structures and the 54,000 decoys with the best shape complementarity score (described below) are kept. The decoys are clustered to reduce redundancy with a cutoff of 10 Å, which usually yields between 2,000 and 6,000 decoys.

Next, models of the entire complex are built by combining pairwise docking decoys and the models are refined using a genetic algorithm (GA), a combinatorial search algorithm. Multi-LZerD represents a multimeric protein complex as a spanning tree (i.e. a connected graph with no cycles), where nodes are proteins and edges are pairwise docking decoys. The initial population of *M* (set to 200) complex models are generated by random combinations of pairwise decoys. Then complex models in the population undergo iterative modification to search for better models that have better fitness scores. Complexes are modified using a GA operation called mutation, where a model has one random edge (pairwise docking decoy) removed and one random edge replaced (Fig 1). The modified complexes are assessed for atomic clashes (atom pairs closer than 3 Å) and discarded if the number exceeds a threshold (200). Clustering of the complexes was also performed to reduce redundancy. If clustering decreased the number of complexes below the initial population size *M*, complexes were added back at random to fill the population (except for the final population, which is allowed to have fewer than *M* complexes). Complex fitness was evaluated using a molecular mechanics scoring function (described below) and the population was reduced to the *M* complexes with the best fitness score. This procedure of exploring model conformations with better fitness scores is called a generation. 400 mutations were performed in each generation. Each complex was run for 2000 generations and evaluated for convergence based on the fitness score. If the complex had not converged, the GA was run for an additional 1000 generations. The previous paper showed that most of the cases converged within 1000 generations [33, 43]. The overall procedure mimics a population of protein assembly process. Finally, 200 or fewer models were generated. The algorithm of Multi-LZerD and parameters used were not modified from its original work [33]. Refer to the original paper for more details.

### Scoring functions used for decoy comparison

The ranks of the pairwise decoys that comprise a Multi-LZerD complex model were used to predict assembly order (described in detail below). The pairwise decoys for each subunit pair were ranked by a scoring function, which evaluates the binding energy of the decoys. Here we briefly describe the eight scoring functions used in this work. Multi-LZerD is originally equipped with two scores, a shape-based score and a molecular mechanics-based function. In addition, we benchmarked six knowledge-based statistical scores, DFIRE [44], Dligand [37], ITScorePro [45], GOAP [46], OPUS-PSP [47], and SOAP-PP [48]. These statistical scoring functions have been very successful in various problems in protein structure prediction, such as single protein structure prediction, protein-protein docking, and model quality assessment. The general approach of constructing a knowledge-based statistical scoring function is to use the observed distribution of some feature (e.g. atom pair distance or angles) in a set of known protein structures and normalize the distribution by a reference state. Scoring functions typically differ in the choice of features and the reference state considered.

#### LZerD shape score

LZerD represents a protein with its molecular surface, where anchor points are evenly spread. A decoy is evaluated by the LZerD shape-based scoring function [35], which combines four terms. For the interacting anchor points taken from the interacting proteins, two terms are computed: the angle between the surface normals and the 3DZD correlation of the local surface around the anchor points. The surface normal angle evaluates whether the surfaces are parallel and the 3DZD correlation quantifies the complementary of the surface shapes. These two terms are combined with the buried surface area of the decoy and the excluded volume, which is a penalty term representing atom clashes.

#### LZerD molecular mechanics-based score

The LZerD molecular mechanics score [33] is a linear combination of van der Waals, electrostatics, hydrogen and disulfide bond, solvation, and knowledge-based contact potential terms.

#### DFIRE

DFIRE [44] (Distance-scaled, Finite Ideal gas REference state) is a distance-dependent atom contact potential that considers 167 atom types. It uses a reference state of an ideal gas atom distribution in a finite system.

#### Dligand

Dligand [37] uses the DFIRE reference state to create a statistical energy function for protein complexes.

#### GOAP

GOAP [46] (Generalized Orientation-dependent All-atom Potential) adds an orientation-dependent term to DFIRE to take both distance and orientation into account in evaluating atom contacts.

#### ITScorePro

ITScorePro [45] is a distance-dependent atom contact potential based on 20 atom types. Instead of using a reference state, the pair potentials were iteratively refined to reduce error in protein docking prediction.

#### OPUS-PSP

OPUS-PSP [47] (Potential derived from Side-chain Packing) considers orientation-specific packing interactions of side-chains that are classified into 19 rigid blocks. A repulsive energy term is added to prevent steric clash.

#### SOAP-PP

SOAP-PP [48] (Statistically Optimized Atomic Potential for Protein-Protein interactions) is a statistical potential for protein-protein interaction that considers atom pair distances based on 158 atom types, bond orientation, and relative solvent-accessible surface area. The atom pair distances and bond orientation also consider covalent separation, e.g. how many covalent bonds separate the atoms, how many residues separate the atoms, and whether the atoms are part of the same polypeptide chain.

### Docking order prediction using Path-LZerD

We predict the assembly order of a complex by comparing the ranks of the pairwise decoys that were assembled by Multi-LZerD to obtain the whole complex model. For example, if a model of an A-B-C complex is made up of the A-B decoy with rank 1 and the B-C decoy with rank 125, where pairwise decoys are ranked by a scoring function, the complex is predicted to assemble A-B first, followed by AB bound with C (denoted as AB> ABC). Using the ranks of pairwise decoys is rationalized by the thermodynamic consideration that the population of a decoy is determined by its binding free energy.

The free energy of a binding pose *i* between two subunits, A and B, is defined as the difference between the free energy of the complex and the free energy of the subunits:

$$\begin{array}{c} \Delta G_{bind}^{AB,i}=G_{AB}^{i}-\left(G_{A}+G_{B}\right) \end{array}$$ (1)

where $G_{AB}^{i}$ is the free energy of the *i*th binding pose of the AB complex and *G*_*A*_ and *G*_*B*_ are the free energies of subunits A and B, respectively. Thus, the probability that A and B take the binding pose *i* is

$$\begin{array}{c} p_{AB}^{i}=\frac{e^{-\Delta G_{bind}^{AB,i}/kT}}{\sum_{n}e^{-\Delta G_{bind}^{AB,n}/kT}} \end{array}$$ (2)

where *k* is the Boltzmann constant, *T* is the temperature, and *n* is the index of the binding poses. The probability of a binding pose *j* of B and C, $p_{BC}^{j}$, is computed in the same way.

For a complex with three subunits, ABC, assuming the subunits have equal concentrations, binding pose *i* of AB is more populated than binding pose *j* of BC if $p_{AB}^{i}>p_{BC}^{j}$ and the more populated binding pose will statistically assemble first. Moreover, if we assume that different pairwise complexes have normalization factors (denominator of Eq 2) of the same order and similar energy distributions of binding poses, the ordering of the probabilities, $p_{AB}^{i}>p_{BC}^{j}$, follows from the ordering of the ranks, *rank*(*S*_*i*_) < *rank*(*S*_*j*_). Here, *S*_*i*_ is the score that estimates the binding free energy for binding pose *i* of AB and *rank*(*S*_*i*_) is the rank of the score, where the lowest (i.e. best) score has rank 1. Since the assembly order is predicted by considering pairwise decoys, the binding free energy of a subunit to a subcomplex is approximated by a pairwise interaction, i.e. $\Delta G_{bind}^{AB:C}\approx \Delta G_{bind}^{BC}$ or $\Delta G_{bind}^{AC}$, where $\Delta G_{bind}^{AB:C}$ is the binding free energy of the AB subcomplex binding with the C subunit. The binding free energies of pairwise decoys are estimated by the scoring functions introduced above, which have been successfully used for protein structure prediction and docking [36, 37]. The thermodynamic rationale of the assembly order prediction is made under reasonable assumptions in the same spirit as protein structure prediction. As we demonstrate later, the algorithm shows successful prediction in many cases.

The next choice to make in the prediction procedure is which complex models built by Multi-LZerD to use for the binding energy rank comparison. We used two methods that require knowledge of the native structure (non-blind) and two methods that do not (blind). Both non-blind methods use the metric of root mean square deviation (RMSD) to the native complex structure. We used and compared the following four methods, which use different complex models:

**Low RMSD decoy combination method:** In this method, low RMSD pairwise decoys from LZerD are combined to form a low RMSD complex structure. To generate the model, for each pair of subunits, five decoys with the lowest RMSD are selected. The selected pairs are exhaustively combined to create fully assembled complexes. To assemble complexes, concretely, first we examined the native complex and recorded each contacting pair of subunits using the binding interfaces shown in PISA [49] and visual inspection. These interfaces were treated as edges and all possible spanning trees using those edges were constructed. For each spanning tree, the five lowest RMSD pairwise decoys for each edge were exhaustively combined. The lowest RMSD model out of all combinations of pairwise decoys was selected.

**Lowest RMSD method:** From the final GA generation of Multi-LZerD (200 or fewer models), the model with the lowest RMSD to the native structure was selected.

**Final generation method:** This method belongs to the blind strategy,which does not use the tertiary structure of the target protein complex. All models from the final generation of Multi-LZerD were used to predict the assembly pathway. Each model was given one vote, the assembly pathways were tallied, and the most frequently occurring assembly pathway was predicted. Thus, unlike the first two methods, this method uses many models in the final generation of the GA and does not refer to the native structure. For example, for a complex of three chains, A, B, and C, if the final generation has 200 models, among which 160 models indicate an assembly order of AC> ABC based on their score rank of pairwise decoys, 30 models indicate an AB> ABC order, and the rest indicate BC> ABC, the resulting prediction is AC> ABC because it has the majority of votes.

**Consensus across generations method:** This method belongs to the blind strategy. This method is equivalent to the final generation method, except that votes are tallied from the generation 1000 through the final generation. Since each generation produces up to 200 models, the total number of votes will be up to 200,000. Low RMSD decoy combination and lowest RMSD are non-blind strategies which require knowledge of the native complex structure to compute RMSD. In contrast, final generation and consensus across generations are blind strategies which do not require the native complex structure. Given a model of the complete complex selected by the methods above, the pairwise decoys that make up the complete complex were noted and the ranks and Z-scores of their binding scores were compared. The pairwise decoys were sorted by the score rank and ties were resolved using the Z-score. Assembly was predicted to begin with the pair with the lowest score rank and proceeded in ascending order of score rank. In addition to each individual score, the score ranks were summed to form an additional score. The whole procedure of predicting assembly order (path) using Multi-LZerD models is named Path-LZerD.

### Docking order prediction using buried surface area

In order to compare with the four methods in Path-LZerD, we also predicted assembly order using buried surface area (BSA) in two ways, based on previous work [13, 14]. For all cases, solvent-accessible surface area (SASA) was computed using Naccess [50] considering all atoms in the crystal structure. In the first BSA approach, the pairwise buried surface area is computed for each pair of contacting chains: BSA_*AB*_ = SASA_*A*_ + SASA_*B*_ − SASA_*AB*_, where SASA_*A*_ and SASA_*B*_ are the SASAs of chains A and B alone and SASA_*AB*_ is the SASA of the pairwise complex. The pairs were sorted in descending order of BSA and assembled into a spanning tree, where each node is a protein chain and each edge is a contacting pair. *N* − 1 edges were added where *N* is the number of subunits, but an edge was skipped if it forms a cycle.

In the second BSA approach, instead of computing BSA for pairs of subunits, SASA was computed for all possible subcomplexes (e.g. combinations of [2..*N* − 1] chains that form a connected subgraph). Then, BSA was computed for every possible transition between subcomplexes as the difference between the SASA of the component subcomplexes and the SASA of the new subcomplex, e.g. if A combines with BC, BSA_*A*:*BC*_ = SASA_*A*_ + SASA_*BC*_ − SASA_*ABC*_. The complex was then assembled from its components in descending order of BSA. The former approach will be referred to as pairwise BSA while the latter will be referred to as subcomplex BSA.

## Results

We first discuss overall results on bound docking cases followed by analysis on unbound/modeled structure cases. Then, interesting individual cases are further analyzed. We also mention the computational time taken for computing the docking order prediction.

### Prediction on bound docking cases

The overall assembly order prediction results on bound docking cases using Path-LZerD and BSA are summarized in Table 2. In the table, target protein complexes are classified by the number of chains in the complex. On the left, the lowest RMSD of the complex structure models in the final GA generation of Multi-LZerD is shown. Then, the assembly order prediction results are shown for three non-blind strategies: subcomplex BSA, low RMSD decoy combination, and lowest RMSD. The non-blind methods need the native structure of the complex to predict the assembly order. The right columns show the results of two blind strategies, final generation and consensus across generations, which do not need the native structure of the target.

**Table 2:** Summary of assembly order prediction on bound docking cases.

Chains	PDBID	RMSD (Å)	Non-blind strategies					Blind strategies			
			BSA	Low RMSD decoy		Lowest RMSD		Final generation		Consensus	
				Shape	Sum	OPUS-PSP	Sum	GOAP	Sum	GOAP	Sum
3	**1a0r**	0.85 (3)	**1/1**	**1/1**	**1/1**	**1/1**	**1/1**	**1/1**	**1/1**	**1/1**	**1/1**
	**1ikn**	14.51 (1)	0/1	0/1	0/1	0/1	0/1	**1/1**	**1/1**	**1/1**	**1/1**
	**1vcb**	1.16 (3)	**1/1**	**1/1**	**1/1**	**1/1**	**1/1**	**1/1**	**1/1**	**1/1**	**1/1**
	**2aze**	1.00 (3)	**1/1**	**1/1**	**1/1**	**1/1**	**1/1**	**1/1**	**1/1**	**1/1**	**1/1**
4	**1es7**	1.86 (4)	**2/2**	**2/2**	1/2	**2/2**	**2/2**	**2/2**	**2/2**	**2/2**	**2/2**
	**1gpq**	1.74 (4)	**2/2**	**2/2**	**2/2**	**2/2**	**2/2**	**2/2**	**2/2**	**2/2**	**2/2**
	**2e9x**	9.50 (3)	1/2	0/2	0/2	**2/2**	**2/2**	0/2	0/2	0/2	0/2
	1kf6	22.23 (2)	0/2	0/2	**2/2**	1/2	1/2	1/2	1/2	1/2	1/2
	2bq1	24.27 (1)	**2/2**	**2/2**	1/2	**2/2**	1/2	0/2	0/2	0/2	0/2
	2qsp	18.41 (1)	**2/2**	1/2	1/2	1/2	0/2	**2/2**	**2/2**	**2/2**	**2/2**
	3fh6	35.72 (1)	**2/2**	**2/2**	**2/2**	0/2	1/2	0/2	1/2	1/2	1/2
5	**1hez**	11.73 (2)	**3/3**	0/3	0/3	**3/3**	**3/3**	**3/3**	**3/3**	**3/3**	**3/3**
	**1w88**	4.80 (4)	2/3	**3/3**	1/3	**3/3**	**3/3**	1/3	1/3	1/3	1/3
6	1du3	20.86 (1)	**4/4**	**4/4**	**4/4**	2/4	3/4	3/4	3/4	3/4	3/4
	1rlb	22.99 (1)	**4/4**	**4/4**	**4/4**	**4/4**	3/4	2/4	3/4	3/4	3/4
	1s5b	22.09 (2)	**4/4**	1/4	2/4	**4/4**	**4/4**	3/4	**4/4**	3/4	**4/4**
	3vyt	36.81 (1)	**4/4**	2/4	2/4	1/4	1/4	2/4	2/4	2/4	2/4
	4hi0	40.80 (1)	1/4	3/4	2/4	0/4	2/4	**4/4**	0/4	**4/4**	0/4
	4igc	53.52 (2)	0/4	**4/4**	2/4	0/4	2/4	**4/4**	3/4	**4/4**	3/4
7	3uku	36.60 (3)	2/5	0/5	0/5	2/5	0/5	2/5	1/5	2/5	1/5
	4gwp	48.36 (1)	4/5	1/5	1/5	0/5	0/5	0/5	0/5	0/5	0/5
Total hits out of 21			13 (18)	11 (16)	8 (17)	11 (16)	9 (17)	10 (17)	9 (17)	10 (18)	9 (17)
**Total hits in 9 subset**			7 (8)	6 (6)	4 (6)	8 (8)	8(8)	7 (8)	7 (8)	7 (8)	7 (8)
Total subcomplex hits			42/58	34/58	30/58	32/58	33/58	35/58	32/58	37/58	32/58
**Subcomplex hits in 9 subset**			14/16	10/16	7/16	15/16	15/16	12/16	12/16	12/16	12/16

“RMSD” is the lowest RMSD of all models in the final generation of Multi-LZerD. In the RMSD column, the number in parentheses indicates the largest number of subunits that are assembled within 4 Å RMSD (a value of 1 indicates that no pair was assembled within this cutoff). Non-blind methods require knowledge of the entire complex structure. Results are shown as the number of steps correctly predicted, with perfect pathways in bold. X/Y denotes that X assembly steps correctly predicted out of Y steps in total for assembling the target complex. Each step is a correct subcomplex; e.g. if the assembly order is BC> ABC, there is one step consisting of the BC subcomplex. “BSA” shows the results for the subcomplex BSA strategy. For each LZerD strategy, results are shown for the best single score (e.g. Shape, OPUS-PSP) and the sum of score ranks (Sum). The nine complexes in bold are in the well-predicted target subset (where the models have a low RMSD < 2.0 Å, correct topology, or almost correct with only one subunit misplaced). They are: 1a0r, 1ikn, 1vcb, 2aze, 1es7, 1gpq, 2e9x, 1hez, and 1w88. The two rows of total hits summarize the number of correct predictions. The number in parentheses counts partially correct predictions, i.e. the number of targets which have a non-zero value on the left side of /. The last two rows count the number of correctly predicted subcomplexes, i.e. the sum of the left side of /, relative to the total number of subcomplexes, i.e. the sum of the right side of /.

**Fig 2:**
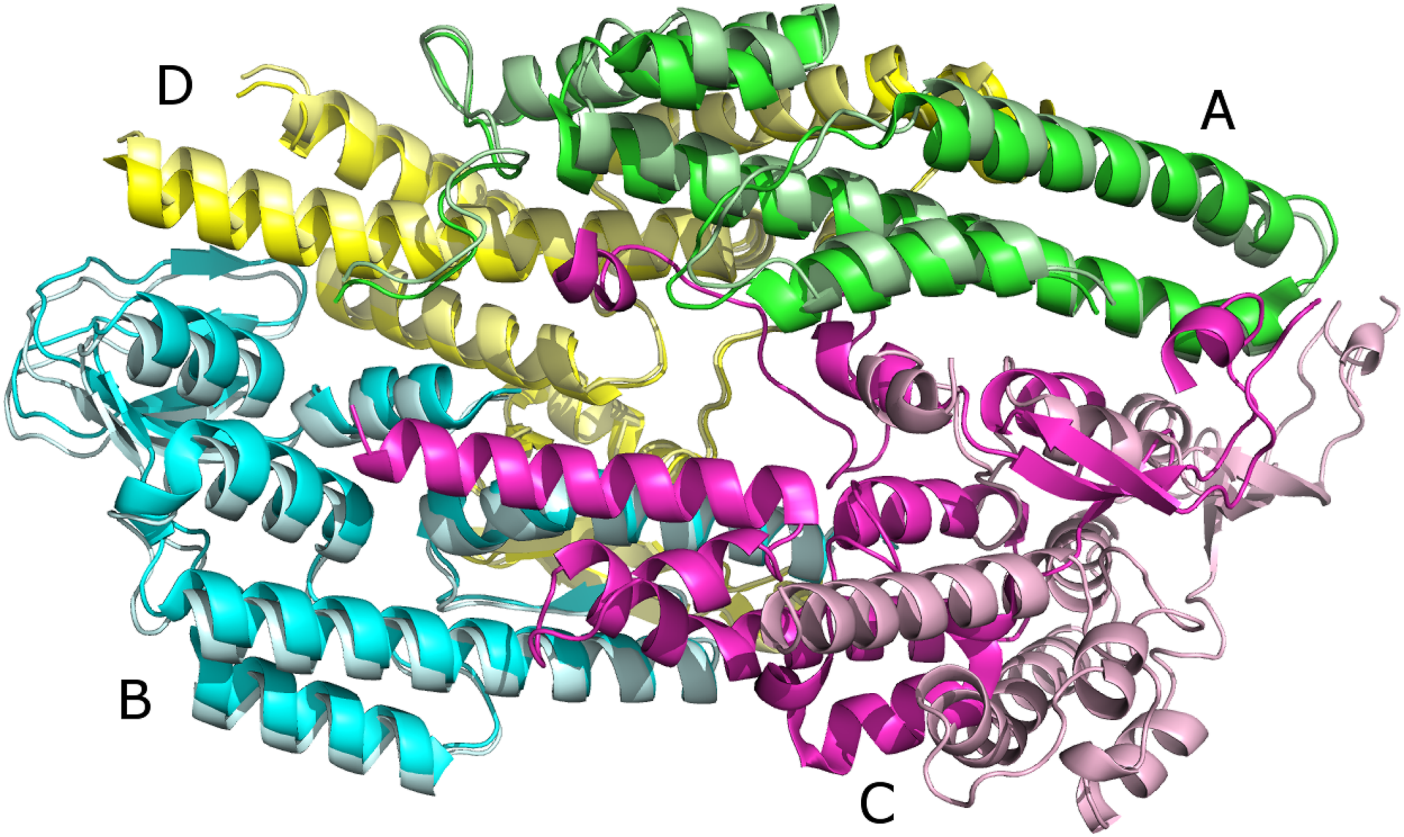
An example of Multi-LZerD prediction that is partially correct. Dark colors: Native structure of 2e9x. Light colors: Multi-LZerD model with 9.5 Å RMSD. Chains A, B, and D (green, cyan, and yellow, respectively) have an RMSD of 1.6 Å. The majority of the RMSD error is due to the position of chain C (magenta).

**Fig 3:**
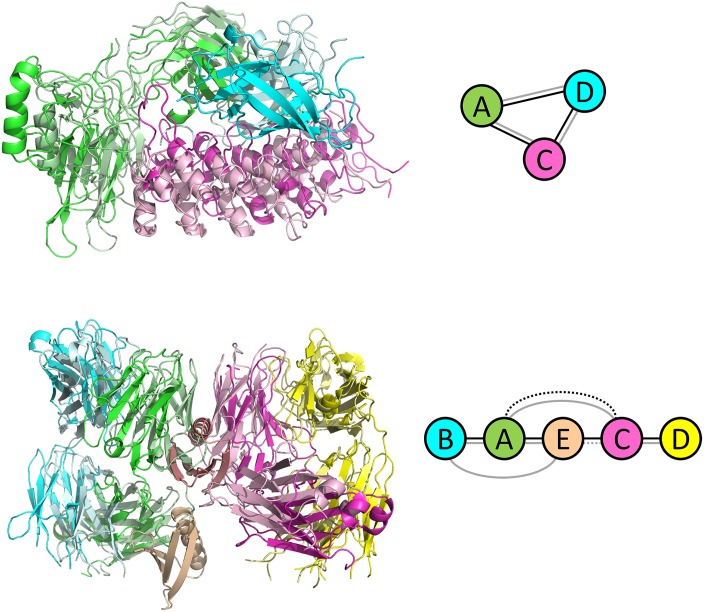
Examples of Multi-LZerD predictions with correct or almost correct topology. Dark colors: native structures. Light colors: lowest RMSD output of Multi-LZerD. Top: 1ikn, 14.51 Å. Bottom: 1hez, 11.73 Å. The diagram to the right of each complex represents the interactions between subunits. Nodes in the diagrams are colored in the same way as the complex structure models. Black lines, interactions in the native structure; gray, the complex model. A solid line indicates that there are more than 20 interacting residue pairs between the subunits and a dotted line is an interaction with fewer than 20 interacting residue pairs. A cutoff distance of 5.0 Å was used to define inter-residue contacts.

Multi-LZerD successfully predicted the structure for many of the complexes but was not able to model the larger 6 and 7 subunit complexes within a small RMSD to the native (Table 2). A model with an RMSD under 2.0 Å was constructed for 5 cases: 1a0r, 1vcb, 2aze, 1es7, and 1gpq. Multi-LZerD usually builds at least a subcomplex structure correctly, even when the overall complex was not correctly assembled [51]. In parentheses is the number of subunits that are assembled within an RMSD of 4.0 Å. Particularly, in two cases, all but one subunit is well predicted (2e9x and 1w88; Fig 2). For the four-chain complex 2e9x, a three-chain subcomplex was assembled with RMSD 1.6 Å and for the five-chain complex 1w88, a four-chain subcomplex was assembled with RMSD 1.3 Å. In another two cases (1ikn and 1hez), the topology was correct or almost correct (Fig 3). The diagram next to each complex illustrates interactions between subunits. Pairwise interfaces are defined as having at least one contacting residue pair (at least one pair of atoms is closer than 5.0 Å.) A solid line in the diagram indicates that there are more than 20 interacting residue pairs between subunits while a dotted line indicates that there are fewer than 20 interacting residue pairs. The lowest RMSD model of 1ikn has the correct topology. The model of 1hez contains all the native interactions with an extra interaction between chain B and E. These nine cases where Multi-LZerD was correct or mostly correct will be referred to as the well-predicted target subset. This subset was also separately analyzed to investigate correlation between the assembly order prediction accuracy and the complex structure prediction accuracy.

Now we turn our attention to the assembly order prediction. A prediction for a target complex is evaluated by the number of correctly predicted assembly steps (X) over the total number of steps (Y) denoted as X/Y. Each step corresponds to a correct subcomplex; for example, if the correct assembly pathway is AB> ABC> ABCD, the predicted pathway AC> ABC> ABCD has a score 1/2 because the second subcomplex is correct. The known assembly steps of each target are shown in Table 1 and a description of the evidence is in S1 Appendix. For each Path-LZerD strategy, the results are shown for the best single score (e.g. shape, OPUS-PSP, GOAP) and for the sum of score ranks. Results for each individual score are shown in supplementary tables, from S2 to S5 Tables. As for prediction using BSA, results for the subcomplex BSA method are shown in Table 2 and the results of the pairwise BSA method are provided in S1 Table.

Overall, the subcomplex BSA method made the largest number of correct predictions when the number of correct full assembly orders was concerned. It correctly predicted the assembly pathway for 13/21 (61.9%) complexes. This is understandable because it is the only method that directly uses the interfaces shown in the native complex structure (note that the two non-blind Path-LZerD methods, the low RMSD and the lowest RMSD methods, refer to the native structure but it is only to identify the lowest RMSD models, which may have substantially different from the native structure). The second was the lowest RMSD decoy and the lowest RMSD strategy by Path-LZerD predicting eleven cases correctly followed by the final generation and the consensus strategy with ten correct predictions.

On the other hand, when the number of partially correctly predicted assembly orders were counted, interestingly, the lowest RMSD method with DFIRE (S3 Table) and the final generation and consensus methods with the molecular mechanics score (S4 and S5 Tables) have the best performance with 19/21(90.5%). This is interesting because the final generation and the consensus methods do not use the native structure to select complex models but still achieved the best performance. In a close look (S4 and S5 Tables), these two Path-LzerD blind strategies made at least partially correct predictions for all but two targets, while the BSA method made three completely wrong predictions. The two blind strategies made partially correct predictions for all the targets with five to seven chains, even though the structure models have large RMSD values.

Among the Path-LZerD strategies, the best performance was observed for the low RMSD decoy combination strategy using the shape score (S2 Table) and the lowest RMSD strategy using OPUS-PSP (S3 Table) when perfect prediction was considered (11/21, 52.4%). If partial correct predictions were counted, the top performing methods were the final generation (S4 Table) and the consensus strategy using molecular mechanics score (S5 Table) (19/21, 90.5%). While the low RMSD decoy combination method has the highest number of perfectly predicted pathways (11/21 using the shape score), it also has the lowest (4/21 using GOAP). The blind methods using Multi-LZerD had a smaller range of numbers: 8-10 perfectly predicted pathways. This suggests that simply choosing pairwise decoys based on RMSD can be either very effective or very ineffective, and that the pairwise decoys that survive the Multi-LZerD genetic algorithm more consistently predict assembly orders probably taking advantage of the voting strategy.

Comparing the BSA method to the Path-LZerD strategies, the assembly pathway of five complexes not predicted by the BSA method were rescued by some of the Path-LZerD strategies: 1ikn, 2e9x, 1kf6, 4hi0, and 4igc. There was only one case where BSA was successful and no LZerD strategy made a perfect prediction: 3vyt. Finally, the assembly pathway of two complexes had no perfect predictions by any method: 3uku and 4gwp.

The success rate of the assembly order prediction of the well-predicted subset of nine complexes (PDB IDs in bold) was higher than for the entire dataset. If only the well-predicted subset of nine complexes is considered, the lowest RMSD strategy with sum of score ranks is more successful than the BSA method with eight out of nine correct predictions. For 4hi0 and 4igc, the BSA method failed to predict their assembly order while Multi-LZerD made successful prediction although the complex structure was not well predicted.

The last two rows of Table 2 evaluate prediction accuracy in a different way by counting the number of correctly predicted subcomplexes that appear during the assembly process. Out of 58 total subcomplexes in all the targets, the BSA method identified 42 (72.4%). The second was the consensus method with GOAP with 37 subcomplexes correctly identified. Interestingly, when methods among Path-LZerD are considered, blind strategies (i.e. the final generation and the consensus method) perform better than the non-blind strategies, having 35 and 37 subcomplex hits. Also, when subcomplexes in the nine target subsets are considered (the last row), Path-LZerD obtained 15 correct subunits, which was better than BSA (14 subunits).

When the results by the final generation method were examined (S4 Table), which involves a voting step by structure models generated in the final generation of Multi-LZerD, it seems that a higher number of votes correlates weakly with assembly path prediction accuracy (S6 Table). For cases with more than 75% of the votes (e.g. 150/200 votes), on average 85.4% of the assembly steps were correctly predicted, while for cases with fewer than 75% of the votes, the average accuracy dropped to 53.1%.

**Fig 4:**
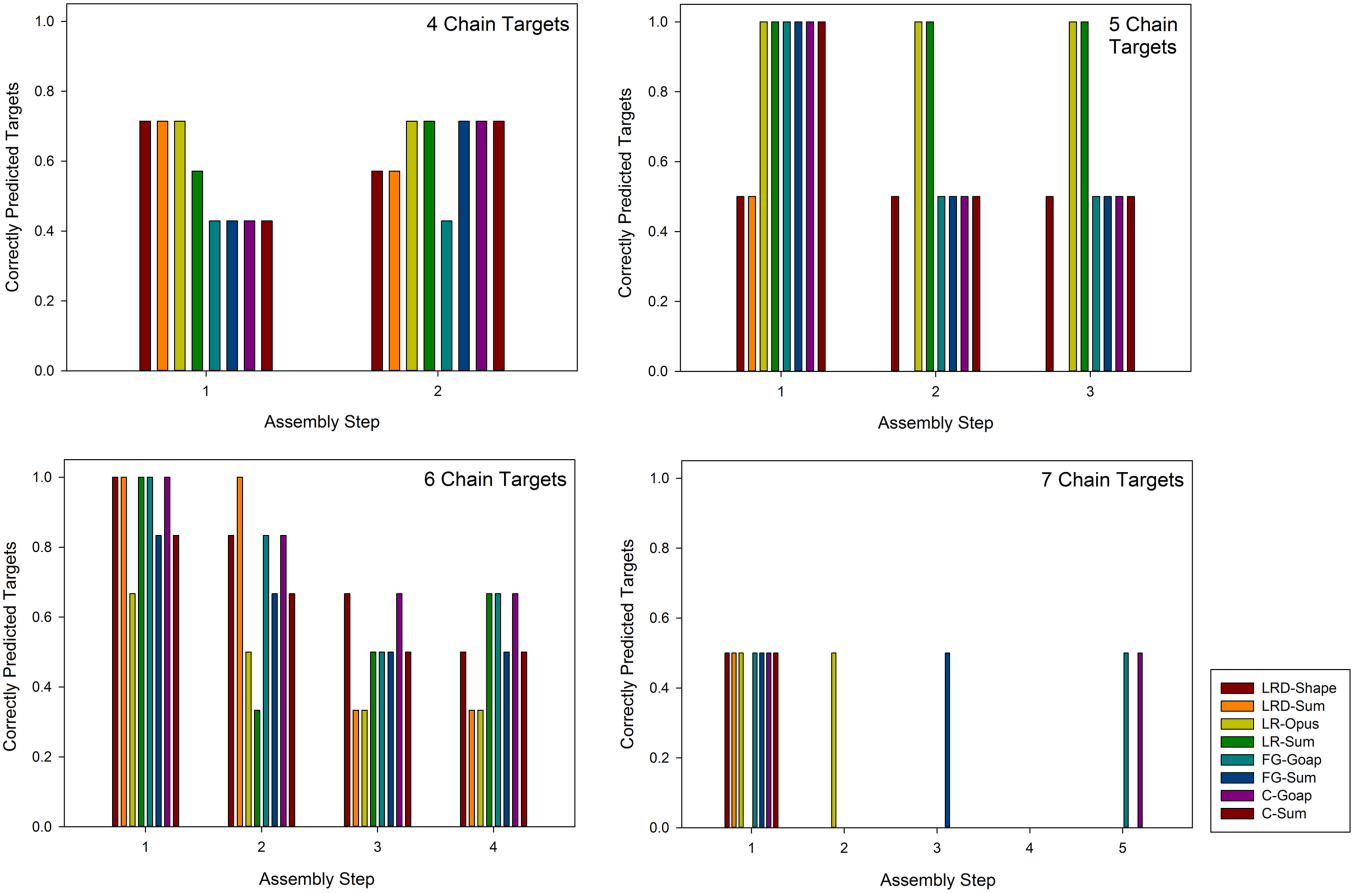
Correctly predicted assembly steps. Targets were classified by the number of chains from four to seven chains (three-chain targets are omitted because there is only one intermediate step) and for each class the number of times each step was correctly predicted was counted. For example, there are six six-chain complex targets in the dataset and a six-chain complex has four intermediate steps (subcomplexes) during the assembly process. The y-axis shows the fraction of targets among the six targets whose particular step was correctly predicted by a prediction method. The color code of the bars shows the assembly prediction methods in Table 2: LRD-Shape and -Sum, the Low RMSD Decoy method with the Shape score and the rank sum; LR-Opus and -Sum, the Lowest RMSD method with OPUS-PSP and rank sum; FG-GOAP and -Sum; the final generation method with GOAP and rank sum; C-GOAP and -Sum, the consensus across generation method with GOAP and rank sum.

We also examined which steps were better predicted in Fig 4. From the plots, the earlier assembly steps, particularly the first step, seem to be better predicted. For the five-chain targets (1hez and 1w88), five methods predicted the first step of both targets correctly, but the second and the third step were predicted for both targets by only two methods. The tendency is clearer for the six-chain targets since there are more assembly steps and targets in this class. The first step of all the targets were correctly predicted by five methods while subsequent steps were less well predicted. For the seven-chain targets, the first step of one out of two targets were correctly predicted by seven methods.

### Prediction on unbound/modeled structure cases

We also predicted the assembly path for unbound cases where individual subunit structures are determined in an isolated condition and cases where subunit structures were computationally modeled. Modeller [52] was used for modeling individual structures from structures of homologous proteins. Homologous proteins to the 21 multimeric protein complexes in Table 1 were searched using BLAST (BLASTP 2.2.31+) runs against protein sequences from the PDB obtained from the Modeller website (https://salilab.org/modeller/supplemental.html). The E-value cutoff used was 0.01. The search found unbound structures for three complexes (1es7, 1rlb, and 3vyt) and template structures for modeling for eight complexes. The results are summarized in Table 3. Only the blind strategies, the final generation method and the consensus across generation method, were used. In the sequence identity (Seq. Id.) column, U indicates that the prediction was made with a complex built from unbound structures while the rest used homology models. For all but three targets, templates with different sequence identity levels were used to see how the quality of models influences assembly path prediction. Detailed information about the individual unbound structures and homology models is provided in S7 Table.

**Table 3:** Summary of the blind strategies on unbound and modeled structure cases.

Chains	PDB ID	Seq Id. (%)	S. RMSD (Å)	RMSD	Final Generation		Consensus	
					GOAP	Sum	GOAP	Sum
3	1a0r	41.7-70.5	1.9-7.4	17.3 (1)	**1/1**	**1/1**	**1/1**	**1/1**
		30.7-52.2	1.9-2.4	**6.3 (2)**	**1/1**	**1/1**	**1/1**	**1/1**
		30.2-37.1	2.4-9.9	**12.7 (1)**	**1/1**	**1/1**	**1/1**	**1/1**
4	1es7	U(4uhz, 2k3g)	1.3-2.3	**10.1 (2)**	**2/2**	**2/2**	**2/2**	**2/2**
		79.8-87.6	0.7-1.1	14.9 (2)	**2/2**	1/2	**2/2**	1/2
		55.3-79.8	1.1-1.3	12.5 (1)	**2/2**	**2/2**	**2/2**	**2/2**
		55.3-58.7	0.8-1.3	12.9 (2)	**2/2**	**2/2**	**2/2**	**2/2**
		28.9-55.3	1.3-9.3	**17.3 (1)**	**2/2**	0/2	**2/2**	**2/2**
	2qsp	87.9-98.6	0.5-0.7	17.2 (2)	0/2	0/2	0/2	0/2
		85.4-87.9	0.6-0.9	19.1 (1)	0/2	0/2	0/2	0/2
	3fh6	27.8-89.1	2.4-6.9	28.6 (1)	1/2	1/2	1/2	1/2
5	1w88	40.8-50.9	0.6-3.3	31.6 (1)	1/3	2/3	0/3	0/3
		40.8-48.7	0.6-3.3	32.2 (1)	0/3	0/3	0/3	0/3
6	1du3	83.2-88.3	1.5-4.3	28.5 (1)	1/4	1/4	1/4	1/4
		35.1-66.3	0.8-2.4	21.8 (1)	1/4	0/4	3/4	1/4
		35.1-39.7	2.3-3.3	24.6 (2)	3/4	3/4	1/4	2/4
	1rlb	U(2nbo, 1kt3)	0.9-11.3	26.0 (1)	0/4	2/4	0/4	0/4
	1s5b	80.3-85.0	0.5-2.1	19.7 (1)	3/4	3/4	3/4	3/4
		60.4-85.0	0.5-0.7	21.8 (1)	3/4	2/4	2/4	1/4
	3vyt	U(2zlc, 2zld, 2zle)	0.9-3.5	42.5 (1)	1/4	1/4	1/4	2/4
7	3uku	33.1-89.4	0.7-12.5	43.0 (1)	0/5	0/5	0/5	0/5
		33.1-69.1	0.7-12.5	36.6 (1)	0/5	2/5	0/5	2/5
Total hits out of 22 complex models					8 (16)	6 (16)	8 (15)	7 (16)
Total hits out of 10 complexes (best hit for each PDB ID)					2 (7)	2 (9)	2 (6)	2 (7)
Total subcomplex hits out of 22 complex models					27/63	26/63	25/63	23/63
Subcomplex hits out of 10 complexes (best hit for each PDB ID)					12/31	16/31	11/31	11/31

Seq. Id. (%) shows the the range of the sequence identity of template structures of each chain that were used for homology modeling. U and PDB IDs in parenthesis indicates that the complex structure was assembled from unbound structures. S. RMSD shows the range of the RMSD of single subunit structures. RMSD is the lowest RMSD observed in the final generation models. In the RMSD column, the number in parentheses indicates the largest number of subunits that are assembled within 4 Å RMSD (1 indicates that no pair was assembled within this cutoff, but at least one subunit is modeled within 4 Å RMSD in the case of homology model cases). RMSD in bold shows that the complex model has a correct topology. Perfect path predictions are shown in bold. Rows for total hits count the number of perfect predictions and the number of partially correct predictions among 22 complex models as well as among 10 protein complexes. For the protein complex results, since all but one complex (1rlb) has multiple models with different subunit structure models modeled using different templates, the best result was considered as the result of the complex.

Compared with the prediction results in Table 2, the path predictions showed little to no deterioration for 1a0r, 1es7, and 3fh6, three complexes with up to four chains. For larger complexes with five chains or more, the number of correctly identified subunits decreased, but for most of the cases and still identified a part of the assembly steps correctly. For 1a0r and 1es7, the quality (i.e. RMSD) of the predicted complex structure was significantly worse than the bound cases in Table 2, but interestingly, the assembly path predictions remained almost perfect. For the 1a0r case, a close look at the assembly paths of individual docking models in the final generation found that the successful prediction was possible because the topology of the structure models were correct despite their large RMSD. For the 1es7 case, the first step of the assembly process, the interaction between chain A and C, was well identified due to their large interface area, which led to the correct path prediction. Thus, for these cases, similar to what was observed in Table 2, the assembly path were correctly predicted even in cases that complex structure itself was not well predicted. Path prediction is often not very sensitive to the quality of individual structure or complex structure models, because the underlying docking simulation often captures affinity of subunits that appears from more coarse-grained features of subunit structures.

On the other hand, we also observed in Table 3 that modeled structure cases were substantially worse than the bound cases. Prediction for 1w88 identified one correct subcomplex in the bound case (Table 2), which decreased to 0 in six out of eight results shown in Table 3. A close examination of the unbound predictions found that the RMSD of the pairwise decoys was worse than the bound cases for 1w88: in the bound case, the average of the best RMSD for 10 pairwise decoys was 1.53 Å, while it worsened to 4.39 Å and 4.71 Å for the two modeled cases (sequence identity ranges of 40.8-50.9% and 40.8-48.7%, respectively). Similar situations were observed for 1rlb and 3uku where path prediction for modeled cases did not identify any correct subcomplexes by some strategies. The average best RMSD of pairwise decoys of the 1rlb bound case was 5.96 Å while it was 10.87 Å for the modeled case. For 3uku, pairwise decoys of the bound case had the average best RMSD of 4.41 Å but it deteriorated to 6.23 Å and 6.58 Å in the two sets of modeled cases. Thus, for these cases, the inaccuracy of the individual models negatively affected the quality of the pairwise decoys. This is one of the fundamental problems of current pairwise protein docking prediction [36]—for improvement, the core pairwise docking algorithm, in this case LZerD, needs to be improved in order to achieve better unbound docking performance.

### Bound case studies

**Fig 5:**
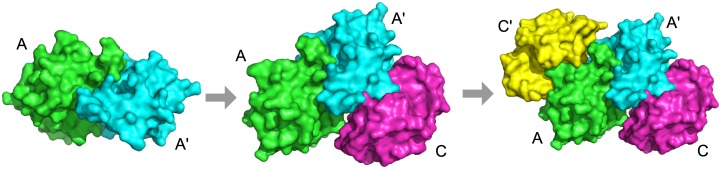
Assembly pathway of 1gpq. Subunits marked A and A′ (green and cyan) are inhibitor of vertebrate lysozyme (IVY) and subunits marked C and C′ (magenta and yellow) are lysozyme C. The assembly pathway is AA′> AA′C> AA′CC′.

We will discuss in detail several complexes with notable results in Table 2. The first complex is 1gpq, which is the complex structure of inhibitor of vertebrate lysozyme (Ivy) from *E. coli* bound to hen egg white lysozyme C. Ivy forms a homodimer (denoted as A and A′ in Fig 5) and binds to lysozyme C (denoted as C and C′). Since Ivy is functional as homodimer, it needs to be formed first. Thus, the known assembly order is AA′> AA′C> AA′CC′ (Fig 5). The structure is predicted correctly at an RMSD of 1.74 Å by Multi-LZerD. The assembly order is predicted perfectly by the BSA method and all Path-LZerD methods. Thus, this is an example where all predictions were correct.

**Fig 6:**
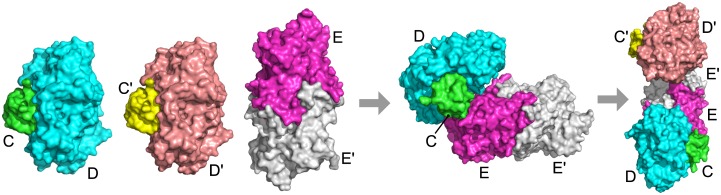
Assembly pathway of 3vyt. Subunits marked C and C′ (green and yellow) are HypC, subunits marked D and D′ (cyan and salmon) are HypD, and subunits marked E and E′ (magenta and white) are HypE. The assembly pathway is CD+C′D′+EE′> CD+C′D′EE′> CC′DD′EE′.

On the other hand, there is one complex that was perfectly predicted by the BSA method but not by Path-LZerD: 3vyt, which is a hexamer comprised of two HypCD heterodimers bound to a central HypE homodimer. HypC (denoted as C and C′ in Fig 6), HypD (D and D′), and HypE (E and E′) are proteins required for the maturation of [NiFe] hydrogenase, which is involved in microbial hydrogen metabolism [53]. Since this complex is an assembly of two HypCD dimers and a HypE homodimer (S1 Appendix Fig 6), the correct assembly order is CD+C′D′+EE′> CD+C′D′EE′> CC′DD′EE′. A problem for this target was that the complex structure was not modeled correctly. The best model had an RMSD of 36.8 Å and no pair of subunits is predicted with RMSD <4.0 Å. Partly due to the incorrect structure model, only part of the order was correctly predicted. For example, the final generation/consensus strategy with the GOAP and the rank sum score correctly predicted the HypE homodimer and one copy of the HypCD heterodimer; however, for higher order subcomplexes, errors emerged. The pathways from the final generation method with the GOAP scoring function are shown in S1 Fig. GOAP gave votes in the final generation to the correct pathway; however, it was a minority (seven). As shown in the diagram, the majority of the pathways correctly identified the EE′ and CD subcomplexes, but D was docked to EE′ without the presence of C for most of the cases, which resulted from underestimation of the strength of the interaction between HypC and HypD.

**Fig 7:**
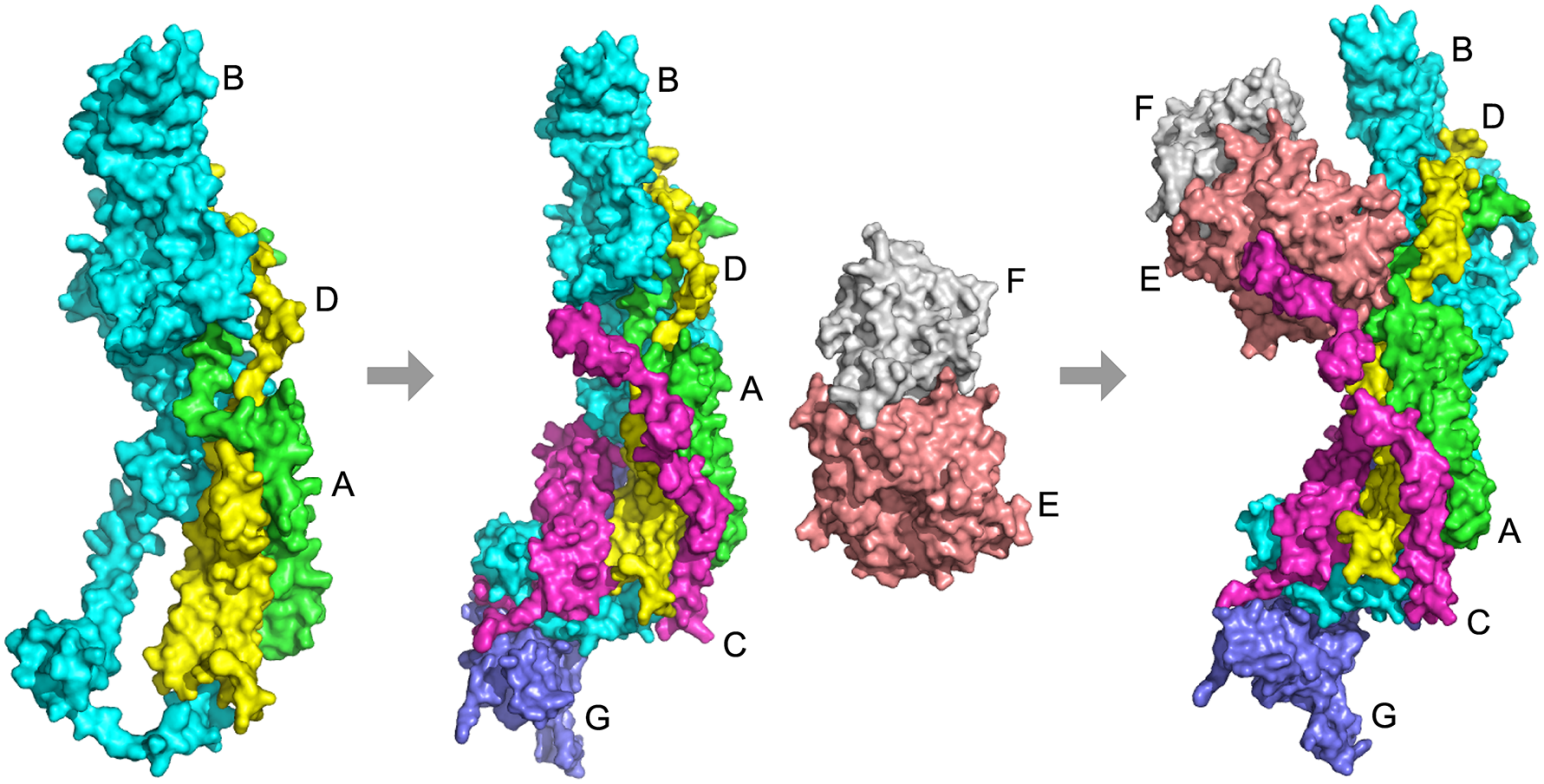
Assembly pathway of 4gwp. Chain A (green) is Med11, chain B (cyan) is Med17, chain C (magenta) is Med8, chain D (yellow) is Med22, chain E (salmon) is Med18, chain F (white) is Med20, and chain G (slate) is Med6. The assembly pathway is ABD> ABCDG+EF> ABCDEFG.

4gwp is an example where Path-LzerD did not perform well. It is the structure of the mediator head module from yeast [54, 55]. Mediator is an essential protein complex that regulates transcription in eukaryotes, connecting activators and repressors that are bound to promoters with RNA polymerase II (Pol II). In yeast, mediator is organized into three modules: head, middle, and tail. The head module plays key roles, including messenger RNA synthesis and interaction with promoters, transcription factor TFIID, and Pol II. The head module is comprised of seven subunits, Med11, Med17, Med8, Med22, Med18, Med20, and Med6. In the PDB file, these subunits correspond to chain A, B, C, D, E, and F, respectively. It is known that the assembly begins with a subcomplex with Med17, Med11, and Med22 (chain A, B, and D), forming a helix bundle. Subsequently, Med8 and Med6 are added (C, G), followed by docking of the Med20–Med18 heterodimer (E, F). Thus, the assembly order is ABD> ABCDG+EF> ABCDEFG (Fig 7). The best output of Multi-LZerD has an RMSD of 34.25 Å and no pair of subunits is predicted within an RMSD below 4.0 Å.It was not trivial for Multi-LZerD to obtain the correct complex structure partly because many subunits have non-compact, elongated conformations and the pairwise decoys do not form tightly packed interactions during the assembly pathway.

The assembly order is predicted almost perfectly by the BSA method, which predicts BD> ABD> ABD+EF> ABD+CG+EF> ABCDG+EF> ABCDEFG. The fourth subcomplex, ABD+CG+EF, is incorrect because chains C and G do not form a dimer before binding [54]. On the other hand, using Path-LZerD, only the low RMSD decoy combination method with some scoring functions obtained partially correct prediction (S2 Table). S2 Fig shows the pathways predicted by the low RMSD decoy combination strategy using DFIRE, GOAP, and the molecular mechanics score. GOAP had both ABD and EF subcomplexes, but also the incorrect CG subcomplex. Both DFIRE and molecular mechanics had the correct steps EF and ABCDG+EF, but not the ABD subcomplex. Although the complete path was not successfully predicted for this complex, it is interesting that the ABD subcomplex, the first subcomplex that appear in the assembly path, and the EF complex, tended to be better captured by the prediction methods. This is consistent with experimental observation that failure of the ABD assembly leads to disassembly of the head [55]. Also, the detection of the EF (Med18–Med20) subcomplex is consistent with their stable hydrophobic interaction, which was detected by various experimental techniques [56–58]. Thus, the path prediction is capturing stable subcomplexes during the assembly process.

**Fig 8:**
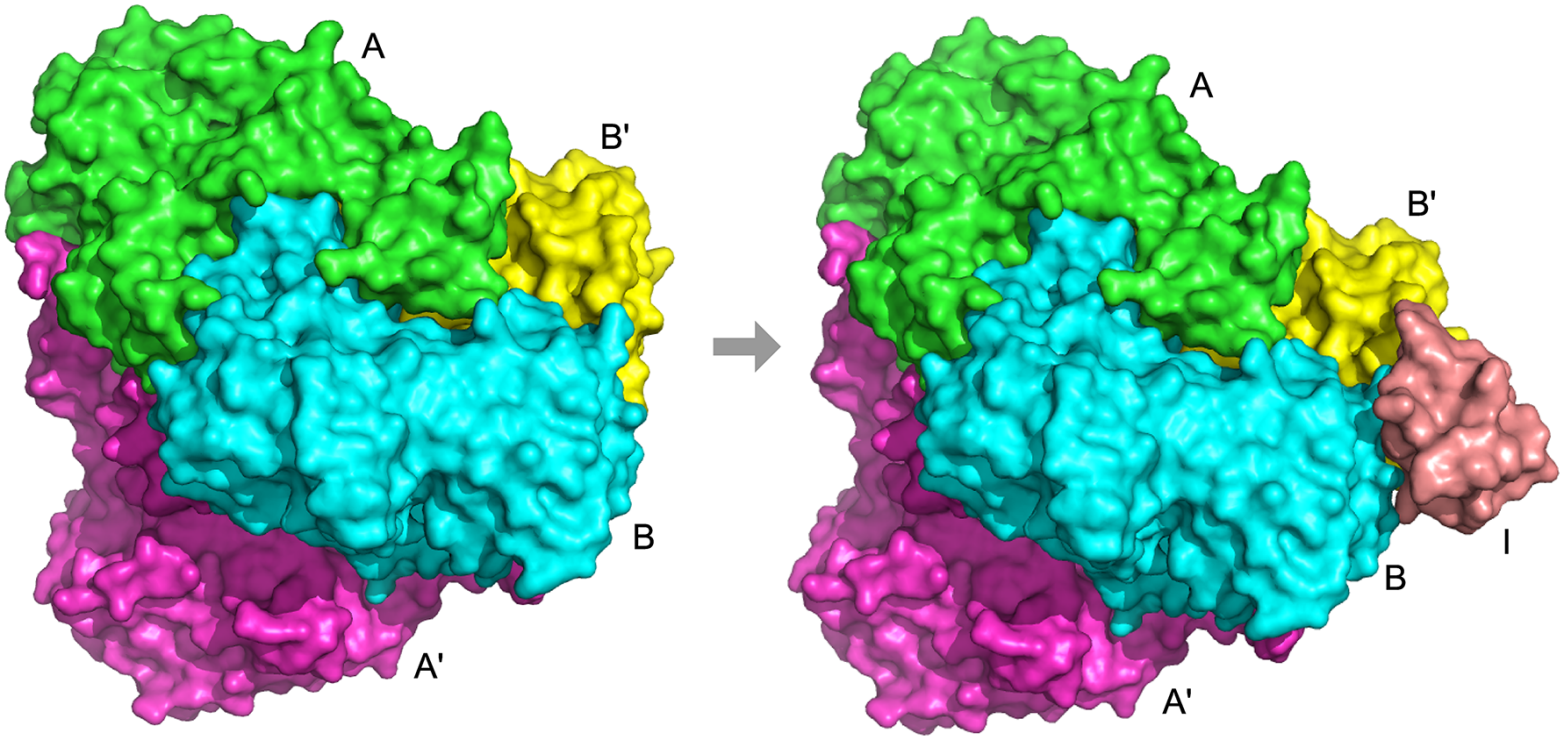
Assembly pathway of 1w88. Subunits marked A and A′ (green and magenta) are pyruvate dehydrogenase E1 *α* subunit, subunits marked B and B′ (cyan and yellow) are E1 *β* subunit, and subunit I (salmon) is the peripheral subunit binding domain of E2. The assembly pathway is AA′BB′> AA′BB′I.

In several cases, the BSA method and Path-LZerD’s non-blind strategies were more successful than the blind strategies. One such case is 1w88, a tetramer of pyruvate dehydrogenase E1 bound to the peripheral subunit binding domain of dihydrolipoyl transacetylase (E2). This complex is part of the pyruvate dehydrogenase multienzyme complex that converts pyruvate into acetyl-CoA, and consists of three enzymes, pyruvate dehydrogenase (E1), dihydrolipoyl transacetylase (E2), and dihydrolipoyl dehydrogenase (E3). The tetramer of E1 with two chains of the *α* subunit and two *β* subunits is expected to form before binding to the E2 subunit (chain I), making the assembly order AA′BB′> AA′BB′I (Fig 8). Multi-LZerD built the tetramer AA′BB′ correctly with an RMSD of 1.3 Å, but misplaced Chain I, which resulted in an overall RMSD of 4.8 Å. Despite the incorrectly placed subunit, the lowest RMSD model method predicted the entire assembly order perfectly with many of the scoring functions (S3 Table) including the sum of score ranks (Table 2). On the other hand, the blind strategies did not predict the entire pathway correctly. Examining the pathways predicted using the final generation strategy and the sum of score ranks revealed that the majority of models, 174/200, predict the assembly pathway BB′> AI+BB′> A′BB′+AI> AA′BB′I (S3 Fig) partly because many of the models in the final generation have large RMSD values with incorrect topologies. Only one model in the final generation, i.e. the lowest RMSD model, was consistent with the correct assembly pathway (S3 Fig). Placing E2 (chain I) in the correct position was difficult in the docking because chain I is very small (49 residues) relative to the other subunits (E1 *α*, A and A′: 368 residues; E1 *β*, B and B′: 324 residues).

**Fig 9:**
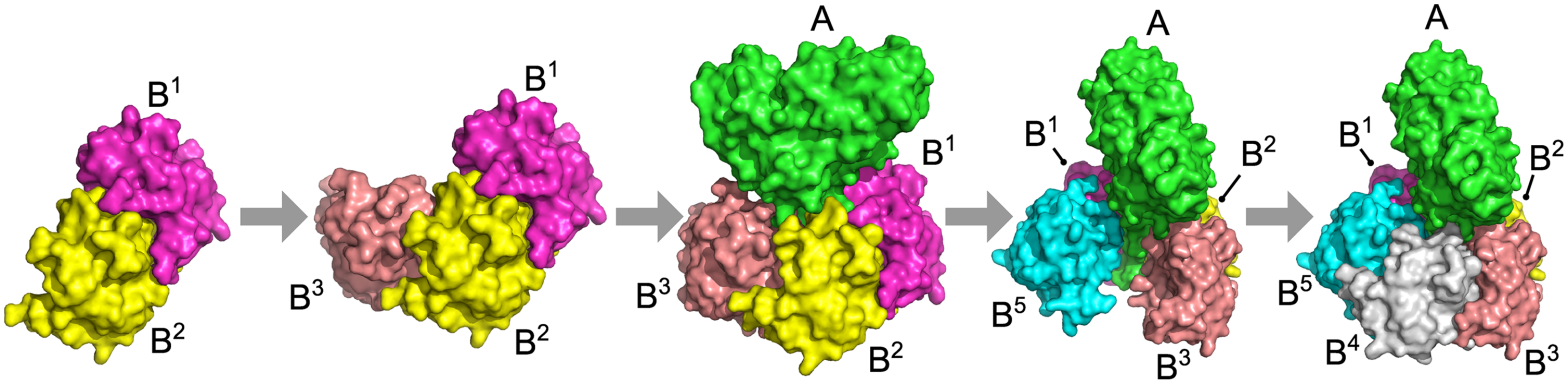
Assembly pathway of 1s5b. Subunit A (green) is the cholera holotoxin A subunit and subunits marked B^1^-B^5^ (cyan, magenta, yellow, salmon, and white) are the cholera holotoxin B subunit. The assembly pathway is B^1^B^2^> B^1^B^2^B^3^> AB^1^B^2^B^3^> AB^1^B^2^B^3^B^4^> AB^1^B^2^B^3^B^4^B^5^.

On the other hand, for some complexes, the overall complex structure was not correctly predicted by Multi-LZerD, but the assembly order prediction was nonetheless successful. 1s5b is such an example. 1s5b is the structure of cholera holotoxin, formed of a homopentamer ring composed of B subunits with the A subunit bound to its face [59]. The B pentamer binds to gangliosides on the surface of target cells while A is an enzyme component, which permanently activates adenylate cyclase. The resulting elevation of cAMP causes ion efflux, leading to severe dehydration. Both components are necessary for in vivo toxic activity. Surprisingly, the homopentamer ring does not form completely prior to the A subunit binding; in fact, if the homopentamer ring is fully assembled in vitro, the A subunit cannot bind [60]. The A subunit binds to a B subunit trimer and forms major contacts with those three subunits [61]; thus, the assembly order is B^1^B^2^> B^1^B^2^B^3^> AB^1^B^2^B^3^> AB^1^B^2^B^3^B^4^> AB^1^B^2^B^3^B^4^B^5^ (Fig 9). The extensive contacts between the A subunit and the B subunit trimer can be seen in the third subcomplex in Fig 9. The homopentamer ring has C5 symmetry formed by five identical homomeric heterologous interfaces. Thus, the pairwise homomeric interfaces have very similar buried surface area (BSA) and therefore, by definition, the pairwise BSA method must predict that all of the interactions form sequentially, e.g. B^1^B^2^> B^1^B^2^B^3^> B^1^B^2^B^3^B^4^> B^1^B^2^B^3^B^4^B^5^> AB^1^B^2^B^3^B^4^B^5^. The assembly pathway exhibited by the cholera holotoxin, in which the ring formation is interrupted by a heteromeric binding step, could never be predicted by the pairwise BSA method (S1 Table). However, the subcomplex BSA method was able to detect that the B subunit trimer forms a larger surface area with the A subunit than the pairwise B interface (Table 2), suggesting that the subunit BSA method is more biologically relevant than the pairwise BSA method.

Despite the fact that the lowest RMSD model of 1s5b produced by Multi-LZerD was 22.09 Å, Path-LZerD, including the blind strategies, predicted the assembly order perfectly using many scoring functions (Table 2, S4 and S5 Tables). Although the correct structure was not predicted for this complex according to the RMSD, 199 out of 200 models in the final generation of docking prediction had almost correct topology with two or fewer additional incorrect subunit interactions, which probably is the main reason of the correct path prediction by the blind strategies. Using OPUS-PSP, the plurality of models, 77, predict the correct assembly pathway in the final generation method (S4 Fig). This suggests that the statistical scoring functions are able to detect the more major contacts that the A subunit makes with three of the B subunits. Knowing the importance of the interaction between chain A and a trimer of B in the assembly process, this protein-protein interaction may be an effective target to block [62] for preventing formation of the whole complex.

The importance of obtaining models with correct topology was also observed for 2qsp, a complex of bovine hemoglobin. The best RMSD of complex models was 18.41 Å; however, the assembly order was correctly predicted by the blind strategies (Table 2). For this target, out of 200 models constructed in the final generation, 147 had almost correct topology with two or fewer extra incorrect subunit interactions, among which 75 (51.0%) voted to the correct assembly order.

**Fig 10:**
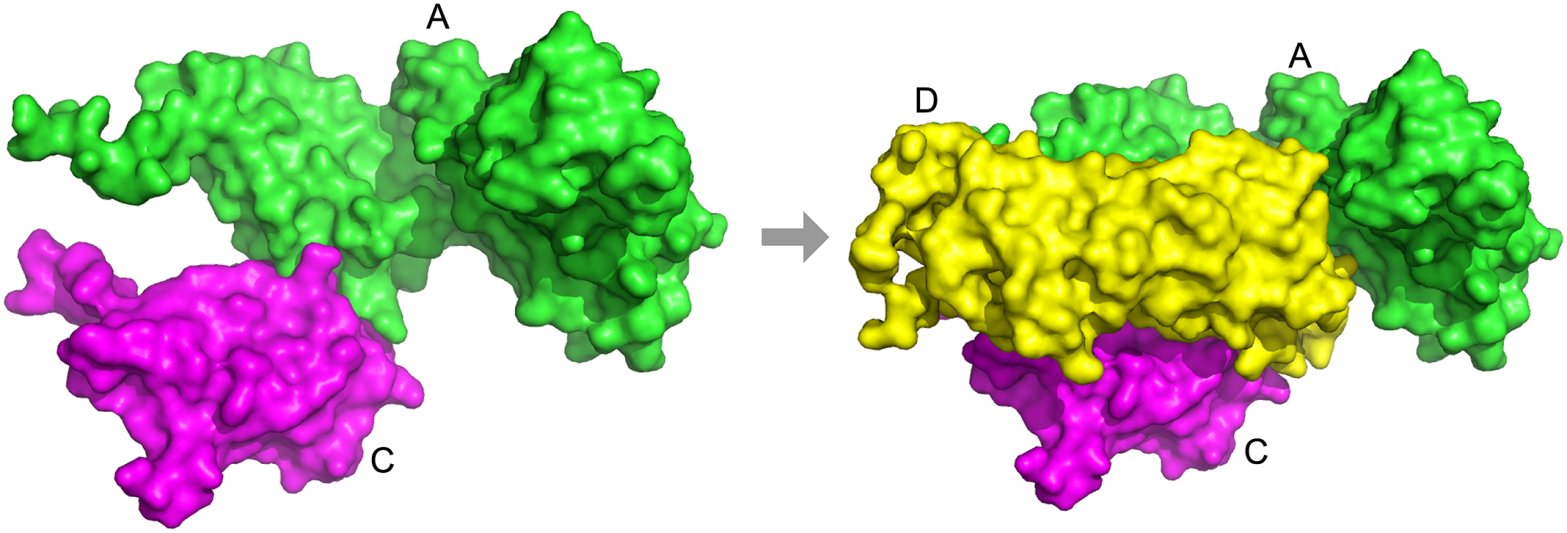
Assembly pathway of 1ikn. Subunit A is p65(RelA), subunit C is p50, and subunit D is I-*κ*-B. The assembly pathway is AC> ACD.

**Fig 11:**
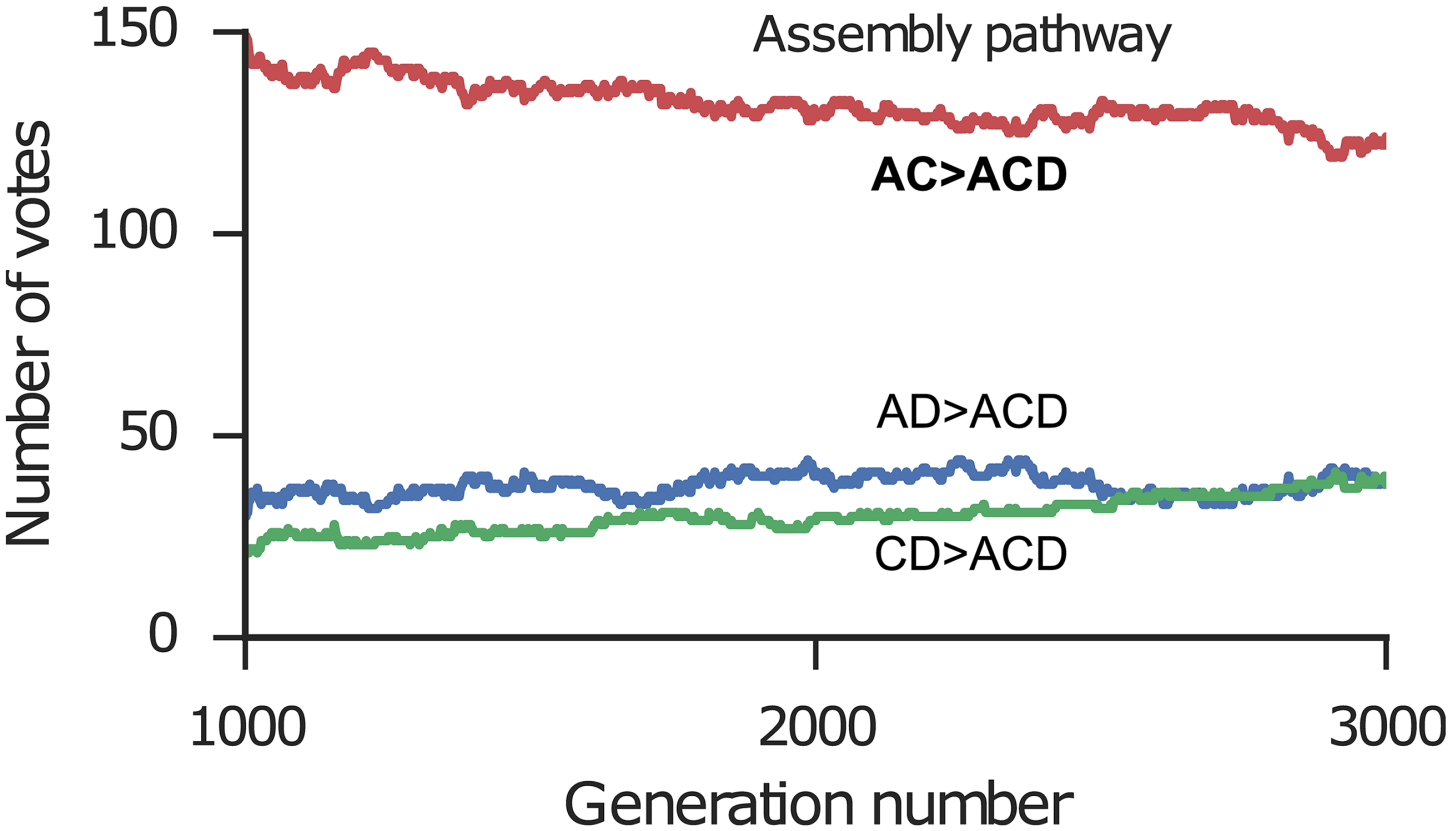
Number of votes for assembly pathways of 1ikn across generations of the genetic algorithm. The pathway for each model is determined using the sum of ranks. The x-axis shows the generation number and the y-axis shows the number of votes for each pathway. Red line and bold label: the correct assembly pathway.

Finally, we discuss two interesting cases where the blind strategies are correct but all of the non-blind strategies including BSA failed. 1ikn is a heterotrimer consisting of p65(RelA)-p50 (an NF-*κ*-B heterodimer) bound to the inhibitor I-*κ*-B. Because the inhibitor binds to the heterodimer, the correct assembly order is AC> ACD (Fig 10). However, the BSA method predicts AD> ADC because the BSA of the AD interface has a larger BSA than the AC interface. The lowest RMSD structure (14.51 Å) has the inhibitor bound primarily to p50; thus, the lowest RMSD methods predict CD> ACD. In contrast, both blind strategies predicted the correct assembly order (Table 2). Using the sum of ranks, the correct pathway had 149 votes in generation 1000 and declined slightly to 124 votes by generation 3000 (Fig 11). Nevertheless, the correct pathway maintained the majority of votes for both the final generation and consensus across generations strategies. In the final generation, 135 out of 200 models show the correct topology (correct connections between subunits using a 5.0 Å cutoff distance), which likely improved prediction accuracy. In this case, despite the lowest RMSD structure being incorrect, the population of multiple models was able to collectively select the correct assembly pathway. It was possible because the knowledge-based scores successfully identified strong interaction between chain A and C despite their smaller interface.

**Fig 12:**
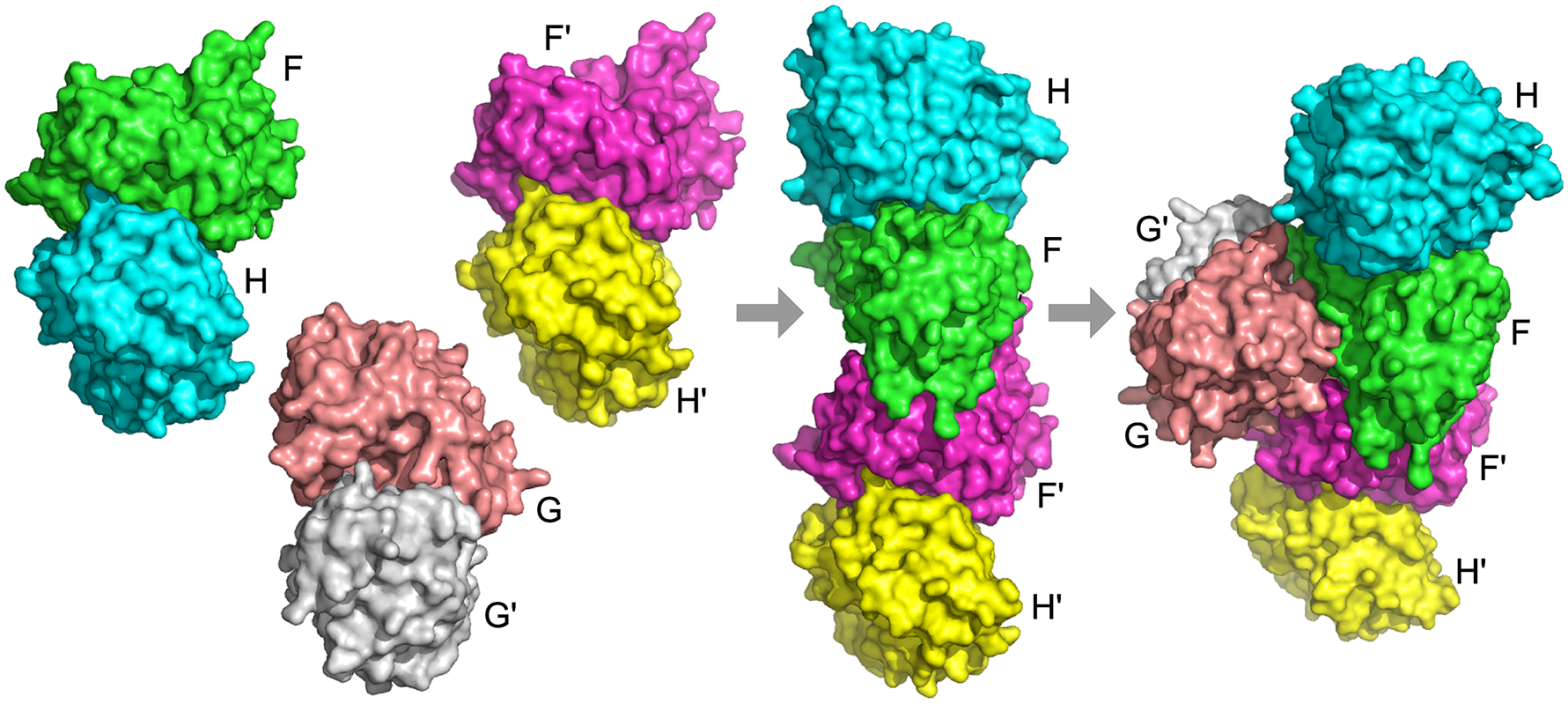
Assembly pathway of 4hi0. Subunits marked F and F′ (green and magenta) are UreF, subunits marked H and H′ (cyan and yellow) are UreH, and subunits marked G and G′ (salmon and white) are UreG. The assembly pathway is FH+F′H′+GG′> FF′HH′+GG′> FF′GG′HH′.

**Fig 13:**
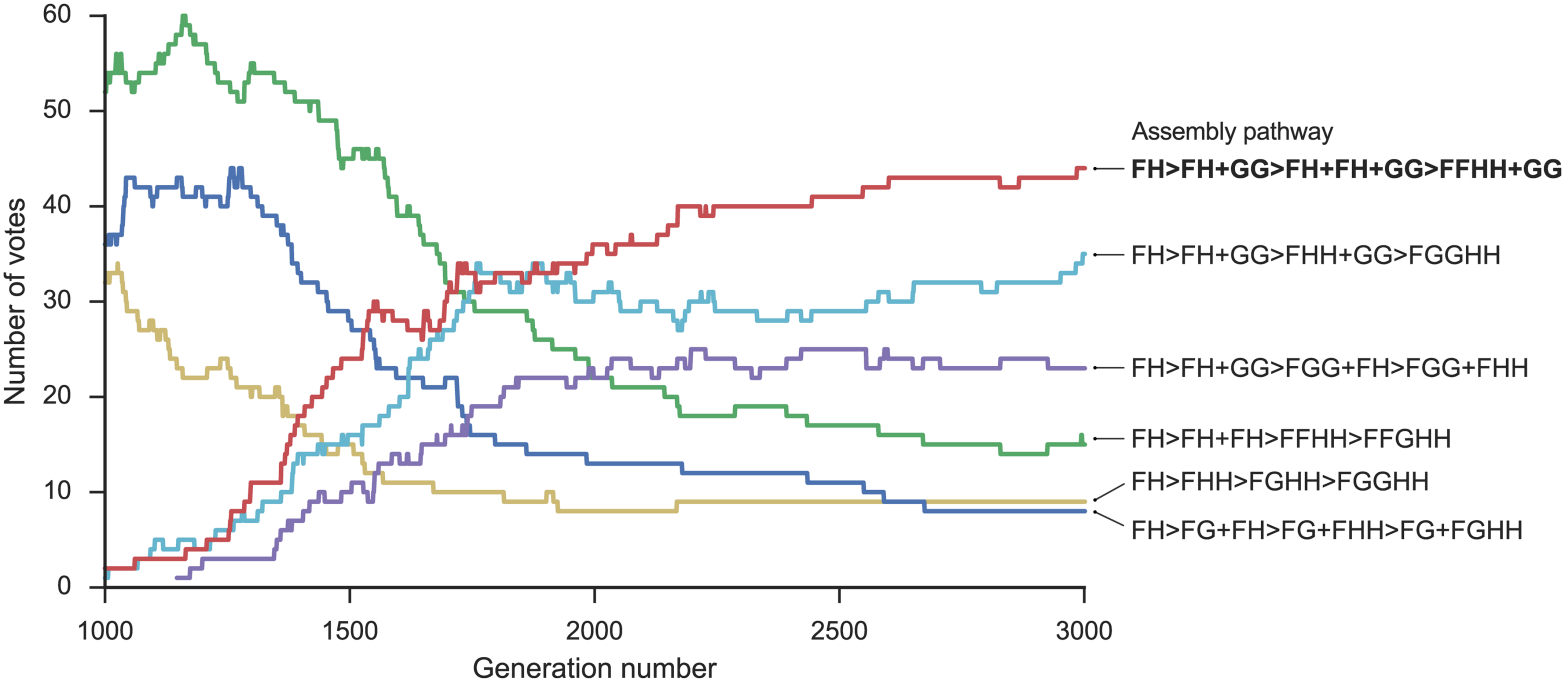
The number of votes for assembly pathways of 4hi0 across generations of the genetic algorithm. The pathway for each model is determined using GOAP. The x-axis shows the generation number and the y-axis shows the number of votes for each pathway. Red line and bold label: the correct assembly pathway. Pathways that received at least 20 votes in at least one generation are shown.

4hi0 is a structure of urease accessory complex from *Helicobacter pylori*, which is involved in maturation of urease. Urease enables the use of urea as the sole nitrogen source is and essential for *H. pylori* to survive in acidic gastric environment. The complex is a hexamer consisting of a dimer of UreF/UreH heterodimers with a UreG homodimer bound. The known assembly order is forming of a dimer of the FH dimer followed by recruiting the G dimer as summarized in S1 Appendix, i.e. FH+F′H′+GG′> FF′HH′+GG′> FF′GG′HH′ (Fig 12). For this complex, the BSA method wrongly predicted that the FF′ interaction occurs first because the FF′ interface is larger than the FH interface. In contrast, for the blind methods, GOAP recognized the strength of the FH interface and perfectly predicts the assembly order (Table 2). In the final generation using GOAP, the plurality of models (44) voted for the correct assembly pathway (S5 Fig). To further explore the process of assembly order prediction with Path-LZerD, we also looked at the number of votes for the correct and incorrect assembly pathways across multiple generations (Fig 13). At generation 1000, the correct pathway had only 2 votes; however, the number of votes increased steadily across 2000 more generations until it achieved plurality. This indicates that the genetic algorithm recognized and rewarded the pairwise interfaces that were consistent with the correct assembly order. To disrupt the formation of this important protein complex for *H. pylori* by a small chemical compound, the target would be the F homodimer, because they have the largest interface and this interaction is prerequisite for recruiting the G dimer, whose GTPase function is essential for urease maturation [63].

These examples demonstrate that there are cases where the largest interface is not the first to form. In such cases, the BSA method will generally fail to predict the correct assembly order, while using scoring functions can lead to successful predictions.

### Computational time

Computational time of the assembly pathway prediction for several examples are shown in Table 4. Path-LZerD has three computational steps: pairwise subunit docking, multimeric complex construction, and path prediction from files from the complex construction process. For a three-chain complex or a small four-chain complex, the total computation was roughly 300 to 500 CPU hours, which is 1 day or less if 20 CPUs (or cores) are used, which is nowadays commonly available. For a five to six chain complex, the time can go up to about 1500 to 2000 CPU hours, which is 3-4 days with 20 CPUs. The time for pairwise docking is essentially proportional to the number of subunit pair combinations (e.g. 3 for a three-chain complex and 15 for a six-chain complex), but the actual time is reduced if there are identical subunits in a complex. The size of proteins is another factor that influences the computational time because in general larger proteins have larger surface area to explore in docking.

**Table 4:** Computational time.

Chains	PDBID	Length	Pair Dock	Multi. Dock	Path	Total
3	1a0r	650	30.5	470.0	2.5	503.0
	1ikn	641	56.0	372.5	2.8	431.3
4	1es7	410	32.0	331.0	1.6	364.6
	2qsp	572	148.3	464.0	5.3	617.6
	3fh6	1552	254.5	1214.5	42.8	1511.8
5	1w88	1433	352.3	1431.3	32.8	1816.4
6	1du3	894	165.0	749.0	5.5	919.5
	1s5b	1303	134.0	624.0	7.3	765.3

Length shows the total length of the chains of a complex. The unit of computational time is in CPU hours. Computational time for a complex is divided into three steps, the pairwise subunit docking (Pair Dock), multiple docking (Multi. Dock; assembling a complex), and computing paths from the docking results (Path). Computations were performed on a computer with Intel Xeon-E5 CPU and 126 or 96 GB RAM.

## Discussion

The assembly order of a protein complex provides not only important insights of the molecular mechanism of complex formation but also useful practical information for obtaining subcomplexes as well as drug and protein design. This is the first systematic study of predicting protein complex assembly order that employed several different approaches. Predicting the assembly order without looking at the experimentally determined structure of the complex is totally new, and is possible by the use of a multimeric protein docking method. For example, it will take too long time for molecular dynamics to simulate an assembly process.

As a core of the algorithm of the assembly pathway prediction, we used our multimeric protein docking program, Multi-LZerD. There are several multimeric docking methods developed in the past. Wolfson and his colleagues pioneered multimeric protein docking with their development of CombDock [64, 65] and their more recent development of DockStar [66]. Methods were also reported that are specific to symmetric multimeric assembly [67–69] and homology-based modeling [70]. Protein docking has extended its applications from structure modeling, which is the original purpose, to other related topics including prediction of protein interactions in a proteome [71, 72] and prediction of protein binding affinity [73]. The current work shows a novel application of the multimeric protein docking algorithm to complex assembly order prediction demonstrating that Multi-LZerD output can be informative even if the quaternary structure and topology are unknown or incorrect. Multi-LZerD generates a pool of models by GA, which turned out to be particularly useful for blind prediction, where a prediction is made without knowing the native structure of the target complex.

In contrast to the BSA method, which needs the experimentally determined quaternary structure of the query protein complex, the current work (Path-LZerD) does not need the structure of the query complex because the method builds the complex structure in the course of assembly order prediction. Rather, it only needs structures of subunits as input for the assembly path prediction, because it performs multimeric protein docking prediction, and examines energy ranking of assembled pairwise decoys (the blind strategies). The results show that the blind strategies worked perfectly for 3-chain targets, and performed well even in some cases where structure of the complexes were not correctly predicted. There are also some cases where the blind prediction worked better than the BSA method. The key observation that led to the development of Path-LZerD was that the pairwise decoy rankings by a binding energy scoring function can indicate docking order of a complex.

Ultimately, it is desired that the approach predicts both the structure and assembly order of a multimeric complex correctly starting from the structures of subunits. Multi-LZerD successfully predicted the structure of a 6-chain complex previously [33] but none of the 6-chain complexes in the assembly order prediction dataset were well predicted (Table 2). Although there were cases where the assembly orders were correctly predicted despite incorrect complex structure modeling, in general the assembly order prediction tends to be more successful when the complex structures are well predicted, as shown for the well-predicted target subset in Table 2. The importance of correctly predicting complex structures is also highlighted in the unbound and the modeled structure cases where the assembly order prediction accuracy deteriorated for larger complexes. Thus, a key for improving the accuracy of docking order prediction is to improve the complex structure prediction. Currently, work is ongoing to improve the performance of Multi-LZerD using more accurate scoring functions [36] and more efficient conformational search methods.

## Supporting information

S1 Appendix Evidence for assembly order. A detailed explanation of the evidence for the assembly order of each complex is provided.
(PDF)

S1 Fig Assembly pathways of 3vyt in the final generation. The pathway for each model is determined using GOAP. Letters in circles indicate subcomplexes, with the complete complex at the bottom. Lines indicate assembly pathways. Numbers indicate how many models vote for each assembly pathway. Assembly pathways with fewer than 7 votes are not visualized. Red line: the correct assembly pathway (CD+C′D′+EE′> CD+C′D′EE′> CC′DD′EE′). Red number: the number of votes for the correct assembly pathway. Blue line: the assembly pathway with the most votes. Blue number: the largest number of votes.
(EPS)

S2 Fig Assembly pathways of 4gwp by low RMSD decoy combination. Letters in circles indicate subcomplexes, with the complete complex at the bottom. Lines indicate assembly pathways. Labels indicate the scoring function used to predict the assembly pathway. Red line: correct assembly pathways (ABD> ABCDG+EF> ABCDEFG).
(EPS)

S3 Fig Assembly pathways of 1w88 in the final generation. The pathway for each model is determined using the sum of score ranks. Letters in circles indicate subcomplexes, with the complete complex at the bottom. Lines indicate assembly pathways. Numbers indicate how many models vote for each assembly pathway. Assembly pathways with fewer than 8 votes are not visualized. Red line: the correct assembly pathway (AA′BB′> AA′BB′I). Red number: the number of votes for the correct assembly pathway. Blue line: the assembly pathway with the most votes. Blue number: the largest number of votes.
(EPS)

S4 Fig Assembly pathways of 1s5b in the final generation. The pathway for each model is determined using OPUS-PSP. Letters in circles indicate subcomplexes, with the complete complex at the bottom. Lines indicate assembly pathways. Numbers indicate how many models vote for each assembly pathway. Lines indicate assembly pathways. Numbers indicate how many models vote for each assembly pathway. Assembly pathways with fewer than 3 votes are not visualized. Red line: the correct assembly pathway (B^1^B^2^> B^1^B^2^B^3^> AB^1^B^2^B^3^> AB^1^B^2^B^3^B^4^> AB^1^B^2^B^3^B^4^B^5^). Red number: the number of votes for the correct assembly pathway.
(EPS)

S5 Fig Assembly pathways of 4hi0 in the final generation. The pathway for each model is determined using GOAP. Letters in circles indicate subcomplexes, with the complete complex at the bottom. Lines indicate assembly pathways. Numbers indicate how many models vote for each assembly pathway. Assembly pathways with fewer than 10 votes are not visualized. Red lines: correct assembly pathways (FH+F′H′+GG′> FF′HH′+GG′> FF′GG′HH′). Red number: the number of votes for the correct assembly pathway.
(EPS)

S1 Table Assembly pathways using the pairwise BSA method and the subcomplex BSA method. (PDF)

S2 Table Assembly pathways using the low RMSD decoy combination strategy. (PDF)

S3 Table Assembly pathways using the lowest RMSD strategy. (PDF)

S4 Table Assembly pathways using the final generation strategy. (PDF)

S5 Table Assembly pathways using the consensus strategy. (PDF)

S6 Table The number of votes in the final generation strategy using GOAP. (PDF)

S7 Table Summary of the unbound structures and template structures used for modeling subunit structures. (PDF)

## References

[1] Yonath A. X-ray crystallography at the heart of life science. Curr Opin Struct Biol. 2011;21(5):622–626. doi: 10.1016/j.sbi.2011.07.005 21824762 10.1016/j.sbi.2011.07.005

[2] Nietlispach D, Mott HR, Stott KM, Nielsen PR, Thiru A, Laue ED. Structure Determination of Protein Complexes by NMR In: Downing AK, editor. Protein NMR Techniques. Totowa, NJ: Humana Press; 2004 p. 255–288. 10.1385/1-59259-809-9:255 15318000

[3] Fiaux J, Bertelsen EB, Horwich AL, Wuthrich K. NMR analysis of a 900K GroEL-GroES complex. Nature. 2002;418(6894):207–211. doi: 10.1038/nature00860 12110894 10.1038/nature00860

[4] Mertens HDT, Svergun DI. Structural characterization of proteins and complexes using small-angle X-ray solution scattering. J Struct Biol. 2010;172(1):128–141. doi: 10.1016/j.jsb.2010.06.012 20558299 10.1016/j.jsb.2010.06.012

[5] Elmlund D, Le SN, Elmlund H. High-resolution cryo-EM: the nuts and bolts. Curr Opin Struct Biol. 2017;46:1–6. doi: 10.1016/j.sbi.2017.03.003 28342396 10.1016/j.sbi.2017.03.003

[6] Esquivel-Rodríguez J, Filos-Gonzalez V, Li B, Kihara D. Pairwise and Multimeric Protein-Protein Docking Using the LZerD Program Suite In: Kihara D, editor. Protein Struct. Predict. vol. 1137 of Methods in Molecular Biology. New York, NY: Springer New York; 2014 p. 209–234. 10.1007/978-1-4939-0366-5_15 24573484

[7] Vakser IA. Protein-Protein Docking: From Interaction to Interactome. Biophys J. 2014;107(8):1785–1793. doi: 10.1016/j.bpj.2014.08.033 25418159 10.1016/j.bpj.2014.08.033 PMC4213718

[8] Park H, DiMaio F, Baker D. The Origin of Consistent Protein Structure Refinement from Structural Averaging. Structure. 2015;23(6):1123–1128. doi: 10.1016/j.str.2015.03.022 25960407 10.1016/j.str.2015.03.022 PMC4456269

[9] Lensink MF, Velankar S, Kryshtafovych A, Huang SY, Schneidman-Duhovny D, Sali A, et al Prediction of homoprotein and heteroprotein complexes by protein docking and template-based modeling: a CASP-CAPRI experiment. Proteins: Struct, Funct, Bioinf. 2016;84(S1):323–348. doi: 10.1002/prot.25007 10.1002/prot.25007 PMC5030136 27122118

[10] Vajda S, Kozakov D. Convergence and combination of methods in protein-protein docking. Curr Opin Struct Biol. 2009;19(2):164–170. doi: 10.1016/j.sbi.2009.02.008 19327983 10.1016/j.sbi.2009.02.008 PMC2763924

[11] Tompa P, Rose GD. The Levinthal paradox of the interactome. Protein Sci. 2011;20(12):2074–2079. doi: 10.1002/pro.747 21987416 10.1002/pro.747 PMC3302650

[12] Levinthal C. How to fold graciously. Mössbauer Spectrosc Biol Syst Proc. 1969;24(41):22–24.

[13] Levy ED, Boeri Erba E, Robinson CV, Teichmann SA. Assembly reflects evolution of protein complexes. Nature. 2008;453(7199):1262–5. doi: 10.1038/nature06942 18563089 10.1038/nature06942 PMC2658002

[14] Marsh JA, Hernandez H, Hall Z, Ahnert SE, Perica T, Robinson CV, et al Protein complexes are under evolutionary selection to assemble via ordered pathways. Cell. 2013;153(2):461–470. doi: 10.1016/j.cell.2013.02.044 23582331 10.1016/j.cell.2013.02.044 PMC4009401

[15] Wells JN, Bergendahl LT, Marsh JA. Operon Gene Order Is Optimized for Ordered Protein Complex Assembly. Cell Rep. 2016;14(4):679–685. doi: 10.1016/j.celrep.2015.12.085 26804901 10.1016/j.celrep.2015.12.085 PMC4742563

[16] Rak M, Gokova S, Tzagoloff A. Modular assembly of yeast mitochondrial ATP synthase. EMBO J. 2011;30(5):920–930. doi: 10.1038/emboj.2010.364 21266956 10.1038/emboj.2010.364 PMC3049208

[17] Mandell DJ, Kortemme T. Computer-aided design of functional protein interactions. Nat Chem Biol. 2009;5(11):797–807. doi: 10.1038/nchembio.251 19841629 10.1038/nchembio.251

[18] Krüger DM, Jessen G, Gohlke H. How Good Are State-of-the-Art Docking Tools in Predicting Ligand Binding Modes in Protein-Protein Interfaces? J Chem Inf Model. 2012;52(11):2807–2811. doi: 10.1021/ci3003599 23072688 10.1021/ci3003599

[19] Tinker JK, Erbe JL, Hol WGJ, Holmes RK. Cholera holotoxin assembly requires a hydrophobic domain at the A-B5 interface: mutational analysis and development of an in vitro assembly system. Infect Immun. 2003;71(7):4093–101. doi: 10.1128/IAI.71.7.4093-4101.2003 12819100 10.1128/IAI.71.7.4093-4101.2003 PMC162025

[20] Friedman FK, Beychok S. Probes of Subunit Assembly and Reconstitution Pathways in Multisubunit Proteins. Annu Rev Biochem. 1979;48(1):217–250. doi: 10.1146/annurev.bi.48.070179.001245 157713 10.1146/annurev.bi.48.070179.001245

[21] Mizushima S, Nomura M. Assembly Mapping of 30S Ribosomal Proteins from E. coli. Nature. 1970;226(5252):1214–1218. doi: 10.1038/2261214a0 4912319 10.1038/2261214a0

[22] Rohl R, Nierhaus KH. Assembly map of the large subunit (50S) of Escherichia coli ribosomes. Proc Natl Acad Sci. 1982;79:729–722. doi: 10.1073/pnas.79.3.729 7038683 10.1073/pnas.79.3.729 PMC345825

[23] Kennedy KA, Gachelet EG, Traxler B. Evidence for multiple pathways in the assembly of the Escherichia coli maltose transport complex. J Biol Chem. 2004;279(32):33290–33297. doi: 10.1074/jbc.M403796200 15192116 10.1074/jbc.M403796200

[24] Heck AJR. Native mass spectrometry: a bridge between interactomics and structural biology. Nat Methods. 2008;5(11):927–933. doi: 10.1038/nmeth.1265 18974734 10.1038/nmeth.1265

[25] Sharon M, Witt S, Glasmacher E, Baumeister W, Robinson CV. Mass Spectrometry Reveals the Missing Links in the Assembly Pathway of the Bacterial 20 S Proteasome. J Biol Chem. 2007;282(25):18448–18457. doi: 10.1074/jbc.M701534200 17430901 10.1074/jbc.M701534200

[26] Bansal PK, Abdulle R, Kitagawa K. Sgt1 Associates with Hsp90: an Initial Step of Assembly of the Core Kinetochore Complex. Mol Cell Biol. 2004;24(18):8069–8079. doi: 10.1128/MCB.24.18.8069-8079.2004 15340069 10.1128/MCB.24.18.8069-8079.2004 PMC515033

[27] Talkington MWT, Siuzdak G, Williamson JR. An assembly landscape for the 30S ribosomal subunit. Nature. 2005;438(7068):628–632. doi: 10.1038/nature04261 16319883 10.1038/nature04261 PMC1444899

[28] Mulder AM, Yoshioka C, Beck AH, Bunner AE, Milligan RA, Potter CS, et al Visualizing Ribosome Biogenesis: Parallel Assembly Pathways for the 30S Subunit. Science. 2010;330(6004):673 LP–677. doi: 10.1126/science.1193220 21030658 10.1126/science.1193220 PMC2990404

[29] Davis JH, Tan YZ, Carragher B, Potter CS, Lyumkis D, Williamson JR. Modular Assembly of the Bacterial Large Ribosomal Subunit. Cell. 2016;167(6):1610–1622.e15 doi: 10.1016/j.cell.2016.11.020 27912064 10.1016/j.cell.2016.11.020 PMC5145266

[30] Hernández H, Robinson CV. Determining the stoichiometry and interactions of macromolecular assemblies from mass spectrometry. Nat Protoc. 2007;2(3):715–726. doi: 10.1038/nprot.2007.73 17406634 10.1038/nprot.2007.73

[31] Chen J, Sawyer N, Regan L. Protein-protein interactions: General trends in the relationship between binding affinity and interfacial buried surface area. Protein Sci. 2013;22:510–515. doi: 10.1002/pro.2230 23389845 10.1002/pro.2230 PMC3610057

[32] Erijman A, Rosenthal E, Shifman JM. How Structure Defines Affinity in Protein-Protein Interactions. PLoS One. 2014;9(10):e110085 doi: 10.1371/journal.pone.0110085 25329579 10.1371/journal.pone.0110085 PMC4199723

[33] Esquivel-Rodríguez J, Yang YD, Kihara D. Multi-LZerD: Multiple protein docking for asymmetric complexes. Proteins: Struct, Funct, Bioinf. 2012;80(7):1818–1833. 10.1002/prot.24079 PMC3370124 22488467

[34] Togawa Y. Prediction of the protein complex assembly pathway using multiple docking algorithm [Master’s thesis]. Purdue University; 2014.

[35] Venkatraman V, Yang YD, Sael L, Kihara D. Protein-protein docking using region-based 3D Zernike descriptors. BMC Bioinformatics. 2009;10:407 doi: 10.1186/1471-2105-10-407 20003235 10.1186/1471-2105-10-407 PMC2800122

[36] Peterson LX, Kim H, Esquivel-Rodríguez J, Roy A, Han X, Shin WH, et al Human and server docking prediction for CAPRI round 30-35 using LZerD with combined scoring functions. Proteins: Struct, Funct, Bioinf. 2017;85(3):513–527. doi: 10.1002/prot.25165 10.1002/prot.25165 PMC5313330 27654025

[37] Zhang C, Liu S, Zhu Q, Zhou Y. A knowledge-based energy function for protein-ligand, protein-protein, and protein-DNA complexes. J Med Chem. 2005;48(7):2325–2335. doi: 10.1021/jm049314d 15801826 10.1021/jm049314d

[38] Berman HM, Westbrook J, Feng Z, Gilliland G, Bhat TN, Weissig H, et al The Protein Data Bank. Nucleic Acids Res. 2000;28(1):235–242. doi: 10.1093/nar/28.1.235 10592235 10.1093/nar/28.1.235 PMC102472

[39] Kihara D, Sael L, Chikhi R, Esquivel-Rodríguez J. Molecular surface representation using 3D Zernike descriptors for protein shape comparison and docking. Curr Protein Pept Sci. 2011;12(6):520–30. doi: 10.2174/138920311796957612 21787306 10.2174/138920311796957612

[40] Li B, Kihara D. Protein docking prediction using predicted protein-protein interface. BMC Bioinformatics. 2012;13(1):7 doi: 10.1186/1471-2105-13-7 22233443 10.1186/1471-2105-13-7 PMC3287255

[41] Canterakis N. 3D Zernike moments and Zernike affine invariants for 3D image analysis and recognition. In: Ersbøll BK, Johansen P, editors. Proc. 11th Scand. Conf. Image Anal. Dansk Selskab for Automatisk Genkendelse af Mønstre; 1999. p. 85–93.

[42] Sael L, La D, Li B, Rustamov R, Kihara D. Rapid comparison of properties on protein surface. Proteins: Struct, Funct, Bioinf. 2008;73(1):1–10. doi: 10.1002/prot.22141 10.1002/prot.22141 PMC2574582 18618695

[43] Esquivel-Rodríguez J, Kihara D. Effect of conformation sampling strategies in genetic algorithm for multiple protein docking. BMC Proc. 2012;6(7):S4 doi: 10.1186/1753-6561-6-S7-S4 23173833 10.1186/1753-6561-6-S7-S4 PMC3504801

[44] Zhou HY, Zhou YQ. Distance-scaled, finite ideal-gas reference state improves structure-derived potentials of mean force for structure selection and stability prediction. Protein Sci. 2002;11(11):2714–2726. doi: 10.1110/ps.0217002 12381853 10.1110/ps.0217002 PMC2373736

[45] Huang SY, Zou X. Statistical mechanics-based method to extract atomic distance-dependent potentials from protein structures. Proteins: Struct, Funct, Bioinf. 2011;79(9):2648–2661. doi: 10.1002/prot.23086 10.1002/prot.23086 PMC11108592 21732421

[46] Zhou H, Skolnick J. GOAP: A generalized orientation-dependent, all-atom statistical potential for protein structure prediction. Biophys J. 2011;101(8):2043–2052. doi: 10.1016/j.bpj.2011.09.012 22004759 10.1016/j.bpj.2011.09.012 PMC3192975

[47] Lu M, Dousis AD, Ma J. OPUS-PSP: An Orientation-dependent Statistical All-atom Potential Derived from Side-chain Packing. J Mol Biol. 2008;376(1):288–301. doi: 10.1016/j.jmb.2007.11.033 18177896 10.1016/j.jmb.2007.11.033 PMC2669442

[48] Dong GQ, Fan H, Schneidman-Duhovny D, Webb B, Sali A, Tramontano A. Optimized atomic statistical potentials: assessment of protein interfaces and loops. Bioinformatics. 2013;29(24):3158–3166. doi: 10.1093/bioinformatics/btt560 24078704 10.1093/bioinformatics/btt560 PMC3842762

[49] Krissinel E, Henrick K. Inference of Macromolecular Assemblies from Crystalline State. J Mol Biol. 2007;372:774–797. doi: 10.1016/j.jmb.2007.05.022 17681537 10.1016/j.jmb.2007.05.022

[50] Hubbard SJ, Thornton J. Naccess version 2.1.1; 1996. http://www.bioinf.manchester.ac.uk/naccess.

[51] Esquivel-Rodríguez J, Kihara D. Evaluation of multiple protein docking structures using correctly predicted pairwise subunits. BMC Bioinformatics. 2012;13(2):S6 doi: 10.1186/1471-2105-13-S2-S6 22536869 10.1186/1471-2105-13-S2-S6 PMC3377905

[52] Fiser A, Sali A. Modeller: generation and refinement of homology-based protein structure models. Meth Enzymol. 2003;374:461–491. doi: 10.1016/S0076-6879(03)74020-8 14696385 10.1016/S0076-6879(03)74020-8

[53] Watanabe S, Matsumi R, Atomi H, Imanaka T, Miki K. Crystal Structures of the HypCD Complex and the HypCDE Ternary Complex: Transient Intermediate Complexes during [NiFe] Hydrogenase Maturation. Structure. 2012;20(12):2124–2137. doi: 10.1016/j.str.2012.09.018 23123111 10.1016/j.str.2012.09.018

[54] Imasaki T, Calero G, Cai G, Tsai KL, Yamada K, Cardelli F, et al Architecture of the Mediator head module. Nature. 2011;475(7355):240–243. doi: 10.1038/nature10162 21725323 10.1038/nature10162 PMC4109712

[55] Takagi Y, Calero G, Komori H, Brown JA, Ehrensberger AH, Hudmon A, et al Head Module Control of Mediator Interactions. Mol Cell. 2006;23(3):355–364. doi: 10.1016/j.molcel.2006.06.007 16885025 10.1016/j.molcel.2006.06.007

[56] Koh SS, Ansari AZ, Ptashne M, Young RA. An activator target in the RNA polymerase II holoenzyme. Mol Cell. 1998;1(6):895–904. doi: 10.1016/S1097-2765(00)80088-X 9660972 10.1016/s1097-2765(00)80088-x

[57] Kang JS, Kim SH, Hwang MS, Han SJ, Lee YC, Kim YJ. The structural and functional organization of the yeast mediator complex. J Biol Chem. 2001;276(45):42003–42010. doi: 10.1074/jbc.M105961200 11555651 10.1074/jbc.M105961200

[58] Guglielmi B, van Berkum NL, Klapholz B, Bijma T, Boube M, Boschiero C, et al A high resolution protein interaction map of the yeast Mediator complex. Nucleic Acids Res. 2004;32(18):5739–5791. doi: 10.1093/nar/gkh878 10.1093/nar/gkh878 PMC524289 15477388

[59] O’Neal CJ, Amaya EI, Jobling MG, Holmes RK, Hol WGJ. Crystal Structures of an Intrinsically Active Cholera Toxin Mutant Yield Insight into the Toxin Activation Mechanism,. Biochemistry. 2004;43(13):3772–3782. doi: 10.1021/bi0360152 15049684 10.1021/bi0360152

[60] Hardy SJ, Holmgren J, Johansson S, Sanchez J, Hirst TR. Coordinated assembly of multisubunit proteins: oligomerization of bacterial enterotoxins in vivo and in vitro. Proc Natl Acad Sci. 1988;85(19):7109–7113. doi: 10.1073/pnas.85.19.7109 3050987 10.1073/pnas.85.19.7109 PMC282133

[61] Moss J, Iglewski B, Vaughan M, Tu AT, editors. Handbook of Natural Toxins, Volume 8: Bacterial Toxins and Virulence Factors in Disease. CRC Press; 1995.

[62] Shin WH, Christoffer CW, Kihara D. In silico structure-based approaches to discover protein-protein interaction-targeting drugs. Methods. 2017;131:22–32. doi: 10.1016/j.ymeth.2017.08.006 28802714 10.1016/j.ymeth.2017.08.006 PMC5683929

[63] Fong YH, Wong HC, Yuen MH, Lau PH, Chen YW, Wong KB. Structure of UreG/UreF/UreH Complex Reveals How Urease Accessory Proteins Facilitate Maturation of Helicobacter pylori Urease. PLOS Biol. 2013;11(10):e1001678 doi: 10.1371/journal.pbio.1001678 24115911 10.1371/journal.pbio.1001678 PMC3792862

[64] Inbar Y, Benyamini H, Nussinov R, Wolfson HJ. Combinatorial docking approach for structure prediction of large proteins and multi-molecular assemblies. Phys Biol. 2005;2(4):S156–S165. doi: 10.1088/1478-3975/2/4/S10 16280621 10.1088/1478-3975/2/4/S10

[65] Inbar Y, Benyamini H, Nussinov R, Wolfson HJ. Prediction of multimolecular assemblies by multiple docking. J Mol Biol. 2005;349(2):435–447. doi: 10.1016/j.jmb.2005.03.039 15890207 10.1016/j.jmb.2005.03.039

[66] Amir N, Cohen D, Wolfson HJ. DockStar: a novel ILP-based integrative method for structural modeling of multimolecular protein complexes. Bioinformatics. 2015;31(17):2801–2807. doi: 10.1093/bioinformatics/btv270 25913207 10.1093/bioinformatics/btv270

[67] Pierce B, Tong W, Weng Z. M-ZDOCK: a grid-based approach for Cn symmetric multimer docking. Bioinformatics. 2004;21(8):1472–1478. doi: 10.1093/bioinformatics/bti229 15613396 10.1093/bioinformatics/bti229

[68] Degiacomi MT, Dal Peraro M. Macromolecular symmetric assembly prediction using swarm intelligence dynamic modeling. Structure. 2013;21(7):1097–1106. doi: 10.1016/j.str.2013.05.014 23810695 10.1016/j.str.2013.05.014

[69] Popov P, Ritchie DW, Grudinin S. DockTrina: Docking triangular protein trimers. Proteins. 2013;82(1):34–44. doi: 10.1002/prot.24344 23775700 10.1002/prot.24344

[70] Bertoni M, Kiefer F, Biasini M, Bordoli L, Schwede T. Modeling protein quaternary structure of homo- and hetero-oligomers beyond binary interactions by homology. Sci Rep. 2017;7(1):10480 doi: 10.1038/s41598-017-09654-8 28874689 10.1038/s41598-017-09654-8 PMC5585393

[71] Matsuzaki Y, Matsuzaki Y, Sato T, Akiyama Y. In silico screening of protein-protein interactions with all-to-all rigid docking and clustering: an application to pathway analysis. J Bioinform Comput Biol. 2009;7(6):991–1012. doi: 10.1142/S0219720009004461 20014475 10.1142/s0219720009004461

[72] Wass MN, Fuentes G, Pons C, Pazos F, Valencia A. Towards the prediction of protein interaction partners using physical docking. Mol Syst Biol. 2011;7(1). doi: 10.1038/msb.2011.3 21326236 10.1038/msb.2011.3 PMC3063693

[73] Kastritis PL, Bonvin AMJJ. Are scoring functions in protein-protein docking ready to predict interactomes? Clues from a novel binding affinity benchmark. J Proteome Res. 2010;9(5):2216–2225. doi: 10.1021/pr9009854 20329755 10.1021/pr9009854

