# Supplementary material for: Modeling the assembly order of multimeric heteroprotein complexes
Source: PLoS Comput Biol. 2018 Jan 12;14(1):e1005937. doi: 10.1371/journal.pcbi.1005937 (PMC5785014; doi:10.1371/journal.pcbi.1005937)
Supplement: S1 Appendix — A detailed explanation of the evidence for the assembly order of each complex is provided. (PDF) [file pcbi.1005937.s001.pdf]

S1 Appendix

A detailed explanation of the evidence for the assembly order of each complex is provided.

1a0r

Heterotrimeric complex of phosducin/transducin $\beta\gamma$ [1]. Phosducin (chain P) binds to the transducin $\beta\gamma$ dimer (chains B and G) in a regulatory fashion. The association between the transducin β and γ subunits is very strong [1, 2]. Thus, the assembly order is BG>BGP.

1ikn

IκBα/NF-κB complex [3]. This complex is p65(RelA)-p50 (an NF-κ-B dimer) [4] and I-κ-B, an inhibitor that binds to NF-κ-B dimers [5]. In the PDB file, p65 is chain A, p50 is chain C, and I-κ-B is chain D. Thus, the assembly order is AC>ACD.

1vcb

The VHL-elonginC-elonginB structure [6]. This complex is between elongin B and C (chains A and B) and von Hippel-Lindau (VHL) tumor suppressor (chain C). Elongin B and C separately have poor interaction with VHL [7]. Thus, the assembly order is AB>ABC.

2aze

The structure of the Rb C-terminal domain bound to an E2F1-DP1 dimer [8]. The heterodimer between DP-1 and E2F1 (chains A and B) acts as a transcription factor; E2F1 has weak DNA-binding affinity in the absence of DP-1 [9]. Also bound is retinoblastoma-associated protein (Rb; chain C). Thus, the assembly order is AB>ABC.

1es7

The complex between BMP-2 and two BMP receptor IA ectodomains [10]. Chains A and C are bone morphogenic protein 2 (BMP-2). Chains B and D are BMP receptor extracellular domain. BMP-2 forms a disulfide-linked homodimer [11]. The two BMP receptor extracellular domains do not contact each other in the PDB structure. Thus, the assembly order is AA′>AA′B>AA′BB′. (chains that have the same molecule ID in the PDB file are referred to using the first chain ID; e.g. chains A and C are referred to as A and A′).

1gpq

The structure of the inhibitor of C-type lysozyme, Ivy from *E. coli* complexed with its target, hen egg white lysozyme C [12]. Chains A and B are a homodimer of Ivy, thus denoted as A and A′ while chains C and D are lysozyme C. Ivy is functional as a homodimer [13] while lysozyme C can function as a monomer or a dimer [14, 15]. The two subunits of lysozyme C do not contact in the

crystal structure. Thus, the assembly order is AA′>AA′C>AA′CC′ (here two chains of lysozyme are denoted as C and C′, because they are identical chains).

1kf6

E. coli quinol-fumarate reductase with bound inhibitor HQNO [16]. Fumarate reductase consists of two hydrophobic proteins (chains C and D) with an iron-sulfur protein (FrdB; chain B) and flavoprotein (FrdA; chain A) bound. Analysis of the assembly using pulse-chase experiments [17] showed that the hydrophobic proteins (C and D) dimerize first, followed by insertion into the membrane. Next, FrdB and FrdA bind in sequence. Thus, the assembly order is CD>BCD>ABCD.

2bq1

Ribonucleotide reductase class 1b holocomplex R1E,R2F from *Salmonella typhimurium* [18]. This complex is of ribonucleotide-diphosphate reductase 2, α (chains E and F) and β (chains I and J) subunits. The two subunits exist separately as homodimers. Thus, the assembly order is EE′+II′> EE′II′.

2e9x

The crystal structure of human GINS core complex [19]. This complex is composed of DNA replication complex GINS proteins Psf1 and Psf2 (chains A and B) and GINS complex subunit 3 (Psf3; chain C) and GINS complex subunit 4 (Sld5; chain D). Mass spectrometry using methanol to disrupt the complex found two subcomplexes: Psf2-Sld5 and Psf1-Psf2-Sld5. Thus, the assembly order is BD>ABD>ABCD.

2qsp

The structure of bovine hemoglobin solved at pH 5.7 [20]. Hemoglobin is comprised of two α subunits (chains A and C) and two β subunits (chains B and D). Biochemical experiments [21] and ESI-MS [22, 23] have shown that hemoglobin assembles by forming two $\alpha\beta$ heterodimers. Thus, the assembly order is AB>AB+A′B′>AA′BB′.

3fh6

The crystal structure of the resting state maltose transporter from *E. coli* [24]. The maltose transporter is composed of a homodimer of MalK (chains A and B) and a heterodimer of MalF and MalG (chains F and G). MalFG is integral to the membrane while the MalK homodimer is a peripheral cytoplasmic protein. Co-immunoprecipitation (Co-IP) has shown different assembly pathways [25]. The assembly pathways are AA′>AA′F *or* AA′>AA′G *or* AA′+FG>AA′FG.

1hez

The structure of antibody-antigen complex [26]. This is the structure of protein L (chain E) with two immunoglobulins (IGs) bound via the κ light chain (chains A and C). In the PDB file, chains B and D are the IG heavy chains. The IGs are expected to assemble prior to binding protein L. Thus, the assembly order is AB>AB+A′B′>AB+A′B′E>AA′BB′E.

1w88

The crystal structure of pyruvate dehydrogenase E1(D180N, E183Q) bound to the peripheral subunit binding domain of E2 [27]. This structure consists of two copies of the pyruvate dehydrogenase E1 α subunit (chains A and C in the PDB file, denoted as A and A', respectively), two copies of the β subunit (chains B and D, denoted as B and B'), and the peripheral subunit binding domain of E2 (chain I). Because the structure of E1 can be solved in the absence of E2 [28], it is expected that E1 will assemble before E2 binds. Thus, the assembly order is AA′BB′>AA′BB′I.

1du3

The crystal structure of TRAIL-SDR5 [29]. Chains D, E, and F form the homotrimeric tumor necrosis factor-related apoptosis inducing ligand (TRAIL) complex. Bound to the outer edges of the complex is death receptor 5 (chains A, B, and C). When the TRAIL complex binds to the receptor, it leads to apoptosis. Because the receptor chains do not contact each other in the PDB structure and the ligand binding induces the homotrimerization of the receptor [29], it follows that the ligand must assemble first. Thus, the assembly order is DD′>DD′D″>ADD′D″>AA′DD′D″>AA′A″DD′D″.

1rlb

The structure of retinol binding protein complexed with transthyretin [30]. Transthyretin (TTR) is a homotetramer composed of chains A, B, C, and D. One of its functions is to transport retinol binding protein (RBP) [30]. The TTR tetramer has two RBPs bound (chains E and F). Because TTR transports RBP, it is expected that TTR assembles first. TTR itself disassembles into dimers based on subunit tethering experiments [31] Thus, the assembly order is AA′>AA′+A″A‴>AA′A″A‴>AA′A″A‴E>AA′A″A‴EE′.

1s5b

The crystal structure of Cholera holotoxin with an A-subunit Y30S mutation Form 3 [32]. Cholera holotoxin has a homopentamer ring of the B subunit with the A subunit bound to the face of the ring. The A subunit is unable to bind to the assembled pentamer [33]. In addition, the A subunit makes major contacts with three B subunits, suggesting that ABBB is a stable intermediate [34]. Thus, the assembly order is B^1B^2>$B^1B^2B^3$>$AB^1B^2B^3$>$AB^1B^2B^3B^4$>$AB^1B^2B^3B^4B^5$. In the PDB file, the B subunit is chains D, E, F, G, and H and the A subunit is chain A.

3vyt

The crystal structure of the HypC-HypD-HypE complex (form I inward) [35]. Transient interactions between HypC, HypD, and HypE are essential for the maturation of [NiFe] hydrogenase, an enzyme used in microbial hydrogen metabolism [35]. This structure is of the interaction between two HypCD heterodimers with one HypE homodimer. The binding affinity between HypC and HypD is greater than the binding affinity between HypCD and the HypE dimer [35]. In addition, isolated HypC and HypD cannot bind to HypE [35]. Thus, the assembly order is CD+C′D′+EE′> CD+C′D′EE′>CC′DD′EE′. In the PDB file, HypC is chains A and D, HypD is chains B and E, and HypE is chains C and F.

4hi0

The crystal structure of *Helicobacter pylori* urease accessory protein UreF/H/G complex [36]. This structure consists of two UreF/UreH heterodimers and one UreG homodimer. The UreF/UreH heterodimer structure has been solved. UreF forms a homodimer, yielding a tetramer with a linear topology: HFFH. It was also shown that disrupting the UreF homodimer prevents binding of UreG. Thus, the assembly order is FH+F′H′+GG′>FF′HH′+GG′>FF′GG′HH′. In the PDB structure, UreF is chains A and C, UreH is chains B and D, and UreG is chains E and F.

4igc

The crystal structure of *E. coli* RNA polymerase σ70 holoenzyme [37]. Assembly of the bacterial RNA polymerase starts from the $\alpha_2\beta$ heterotrimer [38]. The α_2 dimer and $\alpha_2\beta$ heterotrimer have been identified using pulse labeling, immunoprecipitation, and in vitro assembly [39]. The same work also determined that β' binds next [39] but it was later found using crosslinking that the β' subunit is stabilized by the ω subunit [40]. The σ subunit binds last, known because the $\alpha_2\beta\beta'\omega$ pentamer is the core enzyme and can be reconstituted in vitro [38]. In the PDB structure, chains A and B are subunit α (denoted as A and A′), chain C is subunit β, chain D is subunit β', chain E is subunit ω, and chain X is subunit σ. Thus, the assembly order is AA′>AA′C>AA′C+DE> AA′CDE>AA′CDEX. Note that A and A′ both refer to subunit α while the β and β' subunits are different.

3uku

The structure of Arp2/3 complex with bound inhibitor CK-869 (to be published). Co-IP identified that the ARPC2/ARPC4 heterodimer forms the core of the protein, and other heterodimers, ARPC1/ARPC5 and Arp3/ARPC3, are located at the periphery [41]. The same study found that the heterohexamers lacking either Arp2 or ARPC3 are stable. This biochemical analysis is consistent with the interfaces formed in the crystal structure. Yeast two-hybrid assays were unable to detect interactions involving either Arp2 or Arp3, suggesting they assemble late [42]. Chain A is Arp3, chain B is Arp2, chain C is ARPC1, chain D is ARPC2, chain E is ARPC3, chain F is ARPC4, and chain G is ARPC5. Based on the experimental evidence, the likely assembly order is CG+DF> ACDFG>ABCDEFG.

4gwp

Structure of the Mediator Head Module from *S. cerevisiae* [43]. Assembly of the mediator head begins with the heterotrimer of Med17-Med11-Med22 [44]. Next, Med8 and Med6 bind, followed by the Med20-Med18 heterodimer. The Med17-Med11-Med22 trimer, the Med17-Med11-Med22-Med8-Med6 pentamer, and the Med20-Med18 dimer were identified using co-immunoprecipitation [45]. Chain A is Med11, chain B is Med17, chain C is Med8, chain D is Med22, chain E is Med18, chain F is Med20, and chain G is Med6. Thus, the assembly order is ABD>ABCDG+EF> ABCDEFG.

References

1. Loew A, Ho YK, Blundell T, Bax B. Phosducin induces a structural change in transducin $\beta\gamma$. Structure. 1998;6(8):1007–1019. doi:10.1016/S0969-2126(98)00102-6.

2. Dingus J, Hildebrandt JD. Synthesis and Assembly of G Protein betagamma Dimers: Comparison of In Vitro and In Vivo Studies. In: Dupre DJ, Hebert TE, Jockers R, editors. GPCR Signalling Complexes - Synthesis, Assembly, Trafficking and Specificity. Springer Netherlands; 2012. p. 155–180.

3. Huxford T, Huang DB, Malek S, Ghosh G. The Crystal Structure of the IκBα/NF-κB Complex Reveals Mechanisms of NF-κB Inactivation. Cell. 1998;95(6):759–770. doi:10.1016/S0092-8674(00)81699-2.

4. Gilmore TD. Introduction to NF-κB: players, pathways, perspectives. Oncogene. 2006;25(51):6680–6684. doi:10.1038/sj.onc.1209954.

5. Perkins ND. Integrating cell-signalling pathways with NF-[kappa]B and IKK function. Nat Rev Mol Cell Biol. 2007;8(1):49–62.

6. Stebbins CE. Structure of the VHL-ElonginC-ElonginB Complex: Implications for VHL Tumor Suppressor Function. Science. 1999;284(5413):455–461. doi:10.1126/science.284.5413.455.

7. Duan D, Pause A, Burgess W, Aso T, Chen D, Garrett K, et al. Inhibition of transcription elongation by the VHL tumor suppressor protein. Science. 1995;269(5229):1402–1406. doi:10.1126/science.7660122.

8. Rubin SM, Gall AL, Zheng N, Pavletich NP. Structure of the Rb C-Terminal Domain Bound to E2F1-DP1: A Mechanism for Phosphorylation-Induced E2F Release. Cell. 2005;123(6):1093–1106. doi:10.1016/j.cell.2005.09.044.

9. Bandara LR, Lam EW, Sørensen TS, Zamanian M, Girling R, La Thangue NB. DP-1: a cell cycle-regulated and phosphorylated component of transcription factor DRTF1/E2F which is functionally important for recognition by pRb and the adenovirus E4 orf 6/7 protein. EMBO J. 1994;13(13):3104–14.

10. Kirsch T, Sebald W, Dreyer MK. Crystal structure of the BMP-2-BRIA ectodomain complex. Nat Struct Mol Biol. 2000;7(6):492–496.

11. Scheufler C, Sebald W, Hülsmeyer M. Crystal structure of human bone morphogenetic protein-2 at 2.7 Å resolution1. J Mol Biol. 1999;287(1):103–115. doi:10.1006/jmbi.1999.2590.

12. Abergel C, Monchois V, Byrne D, Chenivesse S, Lembo F, Lazzaroni JC, et al. Structure and evolution of the Ivy protein family, unexpected lysozyme inhibitors in Gram-negative bacteria. Proc Natl Acad Sci. 2007;104(15):6394–6399. doi:10.1073/pnas.0611019104.

13. Monchois V. Escherichia coli ykfE ORFan Gene Encodes a Potent Inhibitor of C-type Lysozyme. J Biol Chem. 2001;276(21):18437–18441. doi:10.1074/jbc.M010297200.

14. Cegielska-Radziejewska R, Leśnierowski G, Kijowski J. Properties and application of egg white lysozyme and its modified preparations a review. Pol J Food Nutr Sci. 2008;58(1):5–10.

15. Maroufi B, Ranjbar B, Khajeh K, Naderi-Manesh H, Yaghoubi H. Structural studies of hen egg-white lysozyme dimer: Comparison with monomer. Biochim Biophys Acta - Proteins Proteomics. 2008;1784(78):1043–1049. doi:10.1016/j.bbapap.2008.03.010.

16. Iverson TM, Luna-Chavez C, Croal LR, Cecchini G, Rees DC. Crystallographic Studies of the Escherichia coli Quinol-Fumarate Reductase with Inhibitors Bound to the Quinol-binding Site. J Biol Chem. 2002;277(18):16124–16130.

17. Latour DJ, Weiner JH. Assembly of Escherichia coli fumarate reductase holoenzyme. Biochem Cell Biol. 1989;67(6):251–259.

18. Uppsten M, Färnegårdh M, Domkin V, Uhlin U. The First Holocomplex Structure of Ribonucleotide Reductase Gives New Insight into its Mechanism of Action. J Mol Biol. 2006;359(2):365–377. doi:10.1016/j.jmb.2006.03.035.

19. Kamada K, Kubota Y, Arata T, Shindo Y, Hanaoka F. Structure of the human GINS complex and its assembly and functional interface in replication initiation. Nat Struct Mol Biol. 2007;14(5):388–396.

20. Aranda R, Cai H, Worley CE, Levin EJ, Li R, Olson JS, et al. Structural analysis of fish versus mammalian hemoglobins: Effect of the heme pocket environment on autooxidation and hemin loss. Proteins: Struct, Funct, Bioinf. 2009;75(1):217–230. doi:10.1002/prot.22236.

21. Bunn HF. Subunit assembly of hemoglobin: an important determinant of hematologic phenotype. Blood. 1987;69(1):1–6.

22. Liu J, Konermann L. Assembly of hemoglobin from denatured monomeric subunits: heme ligation effects and off-pathway intermediates studied by electrospray mass spectrometry. Biochemistry. 2013;52(10):1717–1724.

23. Boys BL, Konermann L. Folding and Assembly of Hemoglobin Monitored by Electrospray Mass Spectrometry Using an On-line Dialysis System. J Am Soc Mass Spectrom. 2007;18(1):8–16. doi:10.1016/j.jasms.2006.08.013.

24. Khare D, Oldham ML, Orelle C, Davidson AL, Chen J. Alternating Access in Maltose Transporter Mediated by Rigid-Body Rotations. Mol Cell. 2009;33(4):528–536. doi:10.1016/j.molcel.2009.01.035.

25. Kennedy KA, Gachelet EG, Traxler B. Evidence for multiple pathways in the assembly of the Escherichia coli maltose transport complex. J Biol Chem. 2004;279(32):33290–33297. doi:10.1074/jbc.M403796200.

26. Graille M, Stura EA, Housden NG, Beckingham JA, Bottomley SP, Beale D, et al. Complex between Peptostreptococcus magnus protein L and a human antibody reveals structural convergence in the interaction modes of Fab binding proteins. Structure. 2001;9(8):679–687.

27. Frank RAW. A Molecular Switch and Proton Wire Synchronize the Active Sites in Thiamine Enzymes. Science. 2004;306(5697):872–876. doi:10.1126/science.1101030.

28. Kato M, Wynn RM, Chuang JL, Tso SC, Machius M, Li J, et al. Structural Basis for Inactivation of the Human Pyruvate Dehydrogenase Complex by Phosphorylation: Role of Disordered Phosphorylation Loops. Structure. 2008;16(12):1849–1859. doi:10.1016/j.str.2008.10.010.

29. Hymowitz SG, Christinger HW, Fuh G, Ultsch M, O'Connell M, Kelley RF, et al. Triggering Cell Death: The Crystal Structure of Apo2L/TRAIL in a Complex with Death Receptor 5. Mol Cell. 1999;4(4):563–571. doi:10.1016/S1097-2765(00)80207-5.

30. Monaco H, Rizzi M, Coda A. Structure of a complex of two plasma proteins: transthyretin and retinol-binding protein. Science. 1995;268(5213):1039–1041. doi:10.1126/science.7754382.

31. Foss TR, Wiseman RL, Kelly JW. The Pathway by Which the Tetrameric Protein Transthyretin Dissociates. Biochemistry. 2005;44(47):15525–15533. doi:10.1021/bi051608t.

32. O'Neal CJ, Amaya EI, Jobling MG, Holmes RK, Hol WGJ. Crystal Structures of an Intrinsically Active Cholera Toxin Mutant Yield Insight into the Toxin Activation Mechanism,. Biochemistry. 2004;43(13):3772–3782. doi:10.1021/bi0360152.

33. Hardy SJ, Holmgren J, Johansson S, Sanchez J, Hirst TR. Coordinated assembly of multisubunit proteins: oligomerization of bacterial enterotoxins in vivo and in vitro. Proc Natl Acad Sci. 1988;85(19):7109–7113.

34. Moss J, Iglewski B, Vaughan M, Tu AT, editors. Handbook of Natural Toxins, Volume 8: Bacterial Toxins and Virulence Factors in Disease. CRC Press; 1995.

35. Watanabe S, Matsumi R, Atomi H, Imanaka T, Miki K. Crystal Structures of the HypCD Complex and the HypCDE Ternary Complex: Transient Intermediate Complexes during [NiFe] Hydrogenase Maturation. Structure. 2012;20(12):2124–2137. doi:10.1016/j.str.2012.09.018.

36. Fong YH, Wong HC, Yuen MH, Lau PH, Chen YW, Wong KB. Structure of UreG/UreF/UreH Complex Reveals How Urease Accessory Proteins Facilitate Maturation of Helicobacter pylori Urease. PLOS Biol. 2013;11(10):e1001678. doi:10.1371/journal.pbio.1001678.

37. Murakami KS. X-ray crystal structure of Escherichia coli RNA polymerase σ70 holoenzyme. J Biol Chem. 2013;288(13):9126–9134.

38. Mathew R, Chatterji D. The evolving story of the omega subunit of bacterial RNA polymerase. Trends Microbiol. 2006;14(10):450–455. doi:10.1016/j.tim.2006.08.002.

39. Ito K, Iwakura Y, Tshihama A. Biosynthesis of RNA polymerase in Escherichia coli. J Mol Biol. 1975;96(2):257–271. doi:10.1016/0022-2836(75)90347-2.

40. Ghosh P, Ishihama A, Chatterji D. Escherichia coli RNA polymerase subunit ω and its N-terminal domain bind full-length β' to facilitate incorporation into the $\alpha 2\beta$ subassembly. Eur J Biochem. 2001;268(17):4621–4627. doi:10.1046/j.1432-1327.2001.02381.x.

41. Gournier H, Goley ED, Niederstrasser H, Trinh T, Welch MD. Reconstitution of Human Arp2/3 Complex Reveals Critical Roles of Individual Subunits in Complex Structure and Activity. Mol Cell. 2001;8(5):1041–1052. doi:10.1016/S1097-2765(01)00393-8.

42. Zhao X, Yang Z, Qian M, Zhu X. Interactions among Subunits of Human Arp2/3 Complex: p20-Arc as the Hub. Biochem Biophys Res Commun. 2001;280(2):513–517. doi:10.1006/bbrc.2000.4151.

43. Robinson PJJ, Bushnell DA, Trnka MJ, Burlingame AL, Kornberg RD. Structure of the Mediator Head module bound to the carboxy-terminal domain of RNA polymerase II. Proc Natl Acad Sci. 2012;109(44):17931–17935. doi:10.1073/pnas.1215241109.

44. Imasaki T, Calero G, Cai G, Tsai KL, Yamada K, Cardelli F, et al. Architecture of the Mediator head module. Nature. 2011;475(7355):240–243. doi:10.1038/nature10162.

45. Takagi Y, Calero G, Komori H, Brown JA, Ehrensberger AH, Hudmon A, et al. Head Module Control of Mediator Interactions. Mol Cell. 2006;23(3):355–364. doi:10.1016/j.molcel.2006.06.007.
