# Supplementary material for: Modeling the assembly order of multimeric heteroprotein complexes
Source: PLoS Comput Biol. 2018 Jan 12;14(1):e1005937. doi: 10.1371/journal.pcbi.1005937 (PMC5785014; doi:10.1371/journal.pcbi.1005937)
Supplement: S1 Table — (PDF) [file pcbi.1005937.s007.pdf]

S1 Table: Assembly pathways using the pairwise BSA method and the subcomplex BSA method

Chains	PDBID	BSA Pairwise	BSA Subcomplex
3	**1a0r**	**1/1**	**1/1**
	**1ikn**	0/1	0/1
	**1vcb**	**1/1**	**1/1**
	**2aze**	**1/1**	**1/1**
4	**1es7**	**2/2**	**2/2**
	**1gpq**	**2/2**	**2/2**
	**2e9x**	1/2	1/2
	1kf6	0/2	0/2
	2bq1	**2/2**	**2/2**
	2qsp	**2/2**	**2/2**
	3fh6	**2/2**	**2/2**
5	**1hez**	**3/3**	**3/3**
	**1w88**	**3/3**	2/3
6	1du3	**4/4**	**4/4**
	1rlb	2/4	**4/4**
	1s5b	2/4	**4/4**
	3vyt	**4/4**	**4/4**
	4hi0	2/4	1/4
	4igc	0/4	0/4
7	3uku	3/5	2/5
	4gwp	4/5	4/5
	Total hits	12 (18)	13 (18)
	Subset hits	7 (8)	6 (8)
	Subcomplex hits	41	42
	Subcomplex hits (Subset)	14	16

Subset hits counts the number of hits among the nine well-predicted targets (their PDB IDs are shown in bold). Subcomplex hits counts the number of correctly identified subcomplexes (i.e. the left part of /) in all the targets. Subcomplex hits (Subset) counts the correct subcopmlexes in the nine well predicted targets.
