# Supplementary material for: Modeling the assembly order of multimeric heteroprotein complexes
Source: PLoS Comput Biol. 2018 Jan 12;14(1):e1005937. doi: 10.1371/journal.pcbi.1005937 (PMC5785014; doi:10.1371/journal.pcbi.1005937)
Supplement: S2 Table — (PDF) [file pcbi.1005937.s008.pdf]

S2 Table: Assembly pathways using the low RMSD decoy combination strategy

Chains	PDBID	DFIRE	Dligand	GOAP	ITScorePro	OPUS-PSP	Mol. Mech.	Shape	SOAP-PP	sum
3	**1a0r**	**1/1**	**1/1**	**1/1**	**1/1**	**1/1**	**1/1**	**1/1**	**1/1**	**1/1**
	**1ikn**	0/1	0/1	0/1	0/1	0/1	0/1	0/1	0/1	0/1
	**1vcb**	**1/1**	**1/1**	**1/1**	**1/1**	**1/1**	**1/1**	**1/1**	**1/1**	**1/1**
	**2aze**	**1/1**	**1/1**	0/1	**1/1**	**1/1**	0/1	**1/1**	**1/1**	**1/1**
4	**1es7**	**2/2**	**2/2**	0/2	**2/2**	0/2	1/2	**2/2**	**2/2**	1/2
	**1gpq**	**2/2**	**2/2**	**2/2**	**2/2**	**2/2**	**2/2**	**2/2**	**2/2**	**2/2**
	**2e9x**	0/2	0/2	1/2	0/2	0/2	0/2	0/2	1/2	0/2
	1kf6	0/2	0/2	**2/2**	0/2	**2/2**	**2/2**	0/2	1/2	**2/2**
	2bq1	**2/2**	**2/2**	1/2	**2/2**	**2/2**	1/2	**2/2**	1/2	1/2
	2qsp	1/2	1/2	1/2	1/2	1/2	1/2	1/2	1/2	1/2
	3fh6	1/2	1/2	1/2	1/2	**2/2**	**2/2**	**2/2**	**2/2**	**2/2**
5	**1hez**	0/3	0/3	0/3	0/3	2/3	0/3	0/3	0/3	0/3
	**1w88**	1/3	2/3	0/3	1/3	1/3	0/3	**3/3**	0/3	1/3
6	1du3	**4/4**	**4/4**	3/4	**4/4**	3/4	**4/4**	**4/4**	**4/4**	**4/4**
	1rlb	3/4	3/4	2/4	3/4	**4/4**	1/4	**4/4**	3/4	**4/4**
	1s5b	1/4	1/4	1/4	1/4	2/4	1/4	1/4	1/4	2/4
	3vyt	1/4	0/4	3/4	1/4	3/4	1/4	2/4	2/4	2/4
	4hi0	3/4	3/4	1/4	3/4	1/4	0/4	3/4	0/4	2/4
	4igc	0/4	0/4	1/4	0/4	2/4	3/4	**4/4**	0/4	2/4
7	3uku	0/5	0/5	0/5	0/5	0/5	0/5	0/5	0/5	0/5
	4gwp	2/5	0/5	0/5	1/5	2/5	2/5	1/5	0/5	1/5
Total hits		7 (15)	7 (14)	4 (14)	7 (15)	8 (17)	6 (14)	11 (16)	7 (14)	8 (17)
Subset hits		5 (6)	5 (6)	3 (4)	5 (7)	4 (6)	3 (4)	6 (6)	5 (6)	4 (6)
Subcomplex hits		26	24	21	25	32	23	34	23	30
S.comp. hits (Subset)		8	9	5	8	8	5	10	8	7
