# Supplementary material for: Modeling the assembly order of multimeric heteroprotein complexes
Source: PLoS Comput Biol. 2018 Jan 12;14(1):e1005937. doi: 10.1371/journal.pcbi.1005937 (PMC5785014; doi:10.1371/journal.pcbi.1005937)
Supplement: S5 Table — (PDF) [file pcbi.1005937.s011.pdf]

S5 Table: Assembly pathways using the consensus strategy

Chains	PDBID	DFIRE	Dligand	GOAP	ITScorePro	OPUS-PSP	Mol. Mech.	Shape	SOAP-PP	sum
3	**1a0r**	**1/1**	**1/1**	**1/1**	**1/1**	**1/1**	**1/1**	**1/1**	**1/1**	**1/1**
	**1ikn**	**1/1**	**1/1**	**1/1**	**1/1**	**1/1**	**1/1**	**1/1**	0/1	**1/1**
	**1vcb**	**1/1**	**1/1**	**1/1**	**1/1**	**1/1**	**1/1**	**1/1**	**1/1**	**1/1**
	**2aze**	**1/1**	**1/1**	**1/1**	**1/1**	**1/1**	**1/1**	**1/1**	**1/1**	**1/1**
4	**1es7**	**2/2**	**2/2**	**2/2**	**2/2**	**2/2**	**2/2**	**2/2**	**2/2**	**2/2**
	**1gpq**	**2/2**	**2/2**	**2/2**	**2/2**	**2/2**	**2/2**	**2/2**	**2/2**	**2/2**
	**2e9x**	0/2	0/2	0/2	0/2	0/2	0/2	0/2	0/2	0/2
	1kf6	1/2	1/2	1/2	1/2	1/2	1/2	1/2	1/2	1/2
	2bq1	0/2	0/2	0/2	0/2	1/2	0/2	0/2	0/2	0/2
	2qsp	**2/2**	**2/2**	**2/2**	**2/2**	**2/2**	**2/2**	**2/2**	**2/2**	**2/2**
	3fh6	1/2	0/2	1/2	1/2	0/2	1/2	0/2	1/2	1/2
5	**1hez**	**3/3**	**3/3**	**3/3**	**3/3**	**3/3**	**3/3**	**3/3**	**3/3**	**3/3**
	**1w88**	1/3	1/3	1/3	1/3	2/3	1/3	1/3	1/3	1/3
6	1du3	2/4	2/4	3/4	3/4	2/4	1/4	3/4	2/4	3/4
	1rlb	3/4	3/4	3/4	3/4	3/4	2/4	3/4	3/4	3/4
	1s5b	**4/4**	**4/4**	3/4	**4/4**	**4/4**	2/4	3/4	**4/4**	**4/4**
	3vyt	1/4	0/4	2/4	1/4	1/4	2/4	1/4	2/4	2/4
	4hi0	1/4	1/4	**4/4**	1/4	0/4	3/4	0/4	1/4	0/4
	4igc	2/4	2/4	**4/4**	3/4	0/4	2/4	2/4	2/4	3/4
7	3uku	1/5	1/5	2/5	1/5	2/5	1/5	0/5	2/5	1/5
	4gwp	0/5	0/5	0/5	0/5	0/5	2/5	0/5	0/5	0/5
	Total hits	9 (18)	9 (16)	10 (18)	9 (18)	9 (16)	8 (19)	8 (15)	8 (17)	9 (17)
	Subset hits	7 (8)	7 (8)	7 (8)	7 (8)	7 (8)	7 (8)	7 (8)	6 (7)	7 (8)
	Subcomplex hits	30	28	37	32	29	31	27	31	32
	S.comp. hits (Subset)	12	12	12	12	13	12	12	11	12
