# Supplementary material for: Modeling the assembly order of multimeric heteroprotein complexes
Source: PLoS Comput Biol. 2018 Jan 12;14(1):e1005937. doi: 10.1371/journal.pcbi.1005937 (PMC5785014; doi:10.1371/journal.pcbi.1005937)
Supplement: S6 Table — (PDF) [file pcbi.1005937.s012.pdf]

S6 Table: The number of votes in the final generation strategy using GOAP

Chains	PDBID	Votes Total	Largest Votes (%)	Correct Steps
3	**1a0r**	123	123 (100)	1.0
	**1ikn**	200	109 (54.5)	1.0
	**1vcb**	154	136 (88.3)	1.0
	**2aze**	31	31 (100)	1.0
4	**1es7**	200	181 (90.5)	1.0
	**1gpq**	200	182 (91.0)	1.0
	**2e9x**	200	125 (62.5)	0.0
	1kf6	200	154 (77.0)	0.5
	2bq1	200	136 (68.0)	0.0
	2qsp	200	60 (30.0)	1.0
	3fh6	200	93 (46.5)	0.0
5	**1hez**	200	200 (100)	1.0
	**1w88**	200	167 (83.5)	0.33
6	1du3	200	107 (53.5)	0.75
	1rlb	200	61 (30.5)	0.5
	1s5b	200	51 (25.5)	0.75
	3vyt	200	57 (28.5)	0.5
	4hi0	200	44 (22.0)	1.0
	4igc	200	28 (14.0)	1.0
7	3uku	200	17 (8.5)	0.4
	4gwp	200	43 (21.5)	0.0

The number of the largest votes and the accuracy of the assembly pathway prediction by the final generation method using GOAP is summarized. The total number of votes, i.e. the number of models in the final generation, is usually 200 but can be smaller because models are clustered by structural similarity. The correct steps shows the fraction of steps that are correctly identified. For example, 1kf6 has 0.5 because 1 step out of 2 steps of the pathway is correctly predicted and 3uku has 0.4 because 2 out of 5 steps were correctly predicted.
