# Supplementary material for: Modeling the assembly order of multimeric heteroprotein complexes
Source: PLoS Comput Biol. 2018 Jan 12;14(1):e1005937. doi: 10.1371/journal.pcbi.1005937 (PMC5785014; doi:10.1371/journal.pcbi.1005937)
Supplement: S7 Table — (PDF) [file pcbi.1005937.s013.pdf]

S7 Table: Summary of the unbound structures and template structures used for modeling subunit structures

Chains	PDB ID	Seq. ID cutoff (%)	Target	Template	Seq. ID (%)	RMSD (Å)
3	1a0r	71	1a0r-B	2pbi-B	52.2	2.1
			1a0r-G	5he3-G	41.7	1.9
			1a0r-P	1b9x-C	70.5	7.4
		53	1a0r-B	2pbi-B	52.2	2.1
			1a0r-G	5he3-G	41.7	1.9
			1a0r-P	2dbc-A	30.7	2.4
		38	1a0r-B	2ymu-B	30.2	2.9
			1a0r-G	5he0-G	37.1	9.9
			1a0r-P	2dbc-A	30.7	2.4
4	1es7	U(4uhz, 2k3g)	1es7-A	4uhz-A	100.0	1.3
			1es7-B	2k3g-A	100.0	2.0
			1es7-C	4uhz-A	100.0	1.5
			1es7-D	2k3g-A	100.0	2.3
		88	1es7-A	4mid-A	79.8	1.1
			1es7-B	2qja-D	87.6	0.7
			1es7-C	4mid-A	79.8	1.1
			1es7-D	2qja-D	87.6	0.7
		80	1es7-A	4mid-A	79.8	1.1
			1es7-B	3evs-C	55.3	1.3
			1es7-C	4mid-A	79.8	1.1
			1es7-D	4evs-C	55.3	1,3
		59	1es7-A	1m4u-L	58.7	0.8
			1es7-B	3evs-C	55.3	1.3
			1es7-C	1m4u-L	58.7	0.8
			1es7-D	3evs-C	55.3	1.3
		56	1es7-A	5tx6-B	28.9	9.3
			1es7-B	3evs-C	55.3	1.3
			1es7-C	5tx6-B	28.9	9.3
			1es7-D	3evs-C	55.3	1.3
	2qsp	99	2qsp-A	1bz1-C	87.9	0.6
			2qsp-B	3cy5-D	98.6	0.5
			2qsp-C	1bz1-C	87.9	0.7
			2qsp-D	3cy5-D	98.6	0.7
		88	2qsp-A	1bz1-C	87.9	0.6
			2qsp-B	3fh9-B	85.4	0.9
			2qsp-C	1bz1-C	87.9	0.7
			2qsp-D	3fh9-B	85.4	0.9

S7 Table: cont'd

Chains	PDB ID	Seq. ID cutoff (%)	Target	Template	Seq. ID (%)	RMSD (Å)
4	3fh6	89	3fh6-A	2awn-A	88.7	4.6
			3fh6-B	2awn-A	88.7	4.5
			3fh6-F	4xtc-M	27.8	6.9
			3fh6-G	3fh6-I	89.1	2.4
5	1w88	51	1w88-A	1umd-A	40.8	3.3
			1w88-B	1umd-D	50.9	1.1
			1w88-C	1umd-A	40.8	3.3
			1w88-D	1umd-D	50.9	1.1
			1w88-I	4qoy-F	48.7	0.6
		49	1w88-A	1umd-A	40.8	3.3
			1w88-B	1qs0-B	46.1	1.3
			1w88-C	1umd-A	40.8	3.3
			1w88-D	1qs0-B	46.1	1.3
			1w88-I	4qoy-F	48.7	0.6
6	1du3	89	1du3-A	1za3-R	88.3	1.5
			1du3-B	1za3-R	88.3	4.3
			1du3-C	1za3-R	88.3	1.5
			1du3-D	1d2q-B	83.2	3.9
			1du3-E	1d2q-B	83.2	3.0
			1eu3-F	1d2q-B	83.2	4.0
		67	1du3-A	5cir-G	66.3	0.8
			1du3-B	5cir-G	66.3	1.2
			1du3-C	5cir-G	66.3	1.1
			1du3-D	3urf-A	35.1	2.3
			1du3-E	3urf-A	35.1	2.4
			1eu3-F	3urf-A	35.1	2.3
		40	1du3-A	4mxw-R	39.7	3.2
			1du3-B	4mxw-R	39.7	3.3
			1du3-C	4mxw-R	39.7	3.3
			1du3-D	3urf-A	35.1	2.3
			1du3-E	3urf-A	35.1	2.4
			1eu3-F	3urf-A	35.1	2.3
	1rlb	U(2nbo, 1kt3)	1rlb-A	2nbo-A	97.6	11.3
			1rlb-B	2nbo-A	97.6	11.3
			1rlb-C	2nbo-A	97.6	11.3
			1rlb-D	2nbo-A	97.6	11.3
			1rlb-E	1kt3-A	99.4	0.9
			1rlb-F	1kt3-A	99.4	0.9

S7 Table: cont'd

Chains	PDB ID	Seq. ID cutoff (%)	Target	Template	Seq. ID (%)	RMSD (Å)
6	1s5b	88	1s5b-A	1lt4-A	87.2	1.0
			1s5b-D	1ltr-H	85.0	0.5
			1s5b-E	1ltr-H	85.0	0.5
			1s5b-F	1ltr-H	85.0	0.5
			1s5b-G	1ltr-H	85.0	0.5
			1s5b-H	1ltr-H	85.0	0.5
		85	1s5b-A	1tii-A	60.4	0.7
			1s5b-D	1ltr-H	85.0	0.5
			1s5b-E	1ltr-H	85.0	0.5
			1s5b-F	1ltr-H	85.0	0.5
			1s5b-G	1ltr-H	85.0	0.5
			1s5b-H	1ltr-H	85.0	0.5
	3vyt	U(2zlc, 2zld, 2zle)	3vyt-A	2z1c-A	100.0	0.9
			3vyt-B	2z1d-A	99.7	1.0
			3vyt-C	2zle-A	100.0	3.5
			3vyt-D	2z1c-A	100.0	3.5
			3vyt-E	2z1d-A	100.0	3.5
			3cyt-F	2z1e-A	100.0	3.5
7	3uku	90	3uku-A	3dwul-B	58.4	5.7
			3uku-B	3ule-B	89.4	0.5
			3uku-C	3dwl-H	40.5	2.8
			3uku-D	3dwl-I	45.9	3.0
			3uku-E	3dwl-J	33.1	12.5
			3uku-F	3dwl-K	69.1	0.7
			3uku-F	3dwl-G	88.8	4.4
		70	3uku-A	3dwl-B	58.6	5.7
			3uku-B	4efh-A	47.1	2.4
			3uku-C	3dwl-H	40.5	2.8
			3uku-D	3dwl-I	45.9	3.0
			3uku-E	3dwl-J	33.1	12.5
			3uku-F	3dwl-K	69.1	0.7
			3uku-F	3dwl-L	33.3	1.9

Unbound structures and template structures used for structure modeling for unbound/modeled targets in Table 3. For a complex target, multiple combinations of templates were used if available. Seq. ID cutoff (%) shows the highest sequence identity between the templates and their native subunits in the model set. RMSD shows the global RMSD of a model to the bound form of the subunit.
